# Childhood Trauma and Psychosocial Stress Affect Treatment Outcome in Patients With Psoriasis Starting a New Treatment Episode

**DOI:** 10.3389/fpsyt.2022.848708

**Published:** 2022-04-25

**Authors:** Gloria-Beatrice Wintermann, Antonie Louise Bierling, Eva M. J. Peters, Susanne Abraham, Stefan Beissert, Kerstin Weidner

**Affiliations:** ^1^Department of Psychotherapy and Psychosomatic Medicine, University Hospital Carl Gustav Carus Dresden, Technische Universitaet Dresden, Dresden, Germany; ^2^Psychoneuroimmunology Laboratory, Department of Psychosomatic Medicine and Psychotherapy, University Hospital Giessen, Gießen, Germany; ^3^Universitätsmedizin Charité, Berlin, Germany; ^4^Department of Dermatology, University Hospital Carl Gustav Carus Dresden, Technische Universitaet Dresden, Dresden, Germany

**Keywords:** psoriasis, systemic therapy, therapy outcome, childhood trauma, perceived stress, anxiety, depression

## Abstract

**Objective:**

Traumatic childhood experiences and psychosocial stress may predispose the evolvement of somatic diseases. Psoriasis is a multifactorial chronic inflammatory skin disease that often associates with current and past stress. Both may entail pathological alterations in major stress axes and a balance shift in the level of T helper type 1 (Th1) and 2 (Th2) cytokines, affecting the development and course of psoriasis. Until now, it is unclear whether traumatic stress experiences during the childhood or current stress are more frequent in psoriatic compared to skin-healthy individuals, and if they interact with treatment outcome.

**Method:**

In a prospective cohort study, the impact of acute and early childhood stress on the course of dermatological treatment were studied in patients with moderate to severe psoriasis (PSO). Patients were examined before (T1) and about 3 months after (T2) the beginning of a new treatment episode. Assessments included clinical outcomes (Psoriasis Area and Severity Index—PASI, Structured Clinical Interview SCID-I) and patient-reported outcomes (PRO) (Childhood Trauma Questionnaire-CTQ, Perceived Stress Scale-PSS, itching/scratching, Dermatology Life Quality Index-DLQI, Hospital Anxiety and Depression Scale, Body Surface Area, Self-Administered PASI).

**Results:**

*N* = 83 PSO patients (median age 53.7, IQR 37.8, 62.5) and *n* = 66 skin-healthy control subjects (HC) (median age 51.5, IQR 33.3, 59.2) participated. PSO had higher CTQ *physical neglect* than HC, as well as higher PRO levels. The positive impact of improved skin on the skin-related quality of life was moderated by the perceived stress. Acute stress at T1 had a positive effect both on the skin severity and the skin-related quality of life. CTQ total closely interacted with baseline psoriasis severity, and was associated with higher improvement from T1 to T2.

**Conclusion:**

One might tentatively conclude, that chronic psychosocial stressors like childhood maltreatment may predispose the manifestation of psoriasis. The latter may be amplified by acute psychological stressors. In addition, the present evidence suggests that systemic therapies work well in PSO, with childhood trauma and acute psychosocial stress. Both should therefore be routinely assessed and addressed in PSO.

## Introduction

Traumatic event exposure has been associated with poor physical health outcomes and the evolvement of various somatic diseases, either mediated or not by posttraumatic stress disorder or other mental disorders (1). As key moderators, an increased central corticotropin-releasing factor (CRF) activity, altered reactivity of the hypothalamic-pituitary-adrenal (HPA)-axis with profound impact on glucocorticoid resistance and immune activation have been identified (2). Chronic stress may go along with a down-regulation of the HPA-axis, an upregulation of the sympathetic-adrenal-medullary (SAM)-responses and stimulates the secretion of pro-inflammatory cytokines [for a review: (3)].

Psoriasis is a chronic-inflammatory skin disease, affecting about 2.5% of the German population, 125 million people worldwide and posing a serious global problem, for both men and women (4, 5). The affected patients often experience itching, stigmatization and shame for their often disfiguring physical appearance, predisposing them for the evolvement of social anxiety (6). About every third patient presents clinically relevant anxiety and depression (7). Consequently, patients suffer from a tremendously decreased health-related quality of life, irrespective from the extent of body surface area involved (8).

In psoriasis, a set of numerous internal and external factors has been identified as triggers for altering interleukin levels, among them psychosocial stressors (9–11). In line, one third of the patients report the first onset, about 66% an exacerbation of psoriasis, after stressful life-events (12, 13). Besides acute, current stressors, also chronic, past stressors may have an impact on the aetiopathogenesis of so-called “psychosomatic skin diseases”, like the psoriasis.

An increased rate of childhood and adulthood negative traumatic experiences was shown in PSO compared to control groups (14–16). This is of specific pathogenic interest, since a number of studies recently showed that traumatic experiences (e.g., sexual, physical abuse or neglect) during one's childhood may determine an individual's stress sensitivity and predispose not only to increased stress perception and psychopathology but also to the exacerbation of chronic-inflammatory diseases (17–19). It can be supposed, that current and past psychosocial stressors may moderate the relationships between psoriasis, disease coping, therapy outcome and the evolvement of mental disorders [for a review: (3)].

Characteristic of PSO are sharply demarcated, erythrosquamous papules and plaques, usually covered with white or silvery scaling (20). As aetiopathogenetic mechanism, a TH1/TH17-driven inflammatory process was identified, with high secretion of pro-inflammatory cytokines such as tumor necrosis factor-alpha (TNF-α), interferon-gamma (IFN-γ), interleukin(IL)-12/23/17 and IL-1β (21, 22). The major pro-inflammatory effector cytokines of psoriasis are linked with an abnormal proliferation and differentiation of the epidermis, forming keratinocytes (23, 24). In addition, comorbid other inflammatory diseases driven by these cytokines are frequent in PSO, among them metabolic syndrome, rheumatoid arthritis, Crohn's disease, ulcerative colitis (25, 26). Following, psoriasis is increasingly being recognized as systemic inflammatory disorder. The recent development of systemic therapy options and biologics neutralize or inhibit these cytokines and have revolutionized the treatment of moderate to severe psoriasis [for systematic reviews and meta-analyses: (27–30)].

Despite significant innovations in the treatment of psoriasis (20), there is still a considerable proportion of non-responding patients (31). It is conceivable that the severity of childhood trauma experience may contribute to treatment non-response in PSO. This may be moderated by an altered HPA-axis reactivity, as has been already shown for patients with depression (32). Also, PSO with high levels of current worrying, which is assumed to be increased after adverse childhood experiences, are more vulnerable to the impact of acute psychosocial stress on their skin lesions, with detrimental effects on pruritus, scratching and treatment outcome (13, 33, 34). Hence, it can be assumed, that childhood trauma may associate with disease severity and psychopathology in PSO, as well as contribute to treatment non-response via an altered acute stress and HPA-axis reactivity (9–11, 32). However, there is a current lack of understanding how former traumatic experiences and current stressors interact with the quality of life, disease severity and finally, how it interacts with treatment outcome in PSO.

Therefore, the first aim of the present study was to assess PSO according to the kind and extent of childhood trauma. Second, we were interested in the impact of the interaction of childhood trauma and current perceived stress with, e.g., clinical measures on the skin health, as assessed before and about 12 weeks after the beginning of a new treatment episode with systemic therapy and/or intensified local therapy. The results may help to sharpen personalized care and allow to carefully assign PSO with adverse childhood experiences and high psychopathological load to the treatment option most effective for them.

## Materials and Methods

### Ethics Statement

The present investigation was performed according to the Declaration of Helsinki on Biomedical Research Involving Human Subjects and was approved by the local Ethic Committee of the Technische Universität Dresden. After description of the complete study protocol participants signed in a written informed consent.

### Study Participants and Design

The naturalistic longitudinal study was conducted at the Department of Dermatology, belonging to a large University hospital with 1.410 beds at the Technische Universität Dresden. Between October 2015 and September 2019, PSO either seen at the outpatient unit during a regular visit or PSO who came to stay on the inpatient ward, were invited to participate. The dermatologic outpatient unit is specialized on the diagnosis and treatment of psoriasis. Stay on the University inpatient ward had the primary aim to begin a new treatment episode, which included the switch to a systemic therapy and/or intensified local therapy, in most cases. PSO had to meet the inclusion and exclusion criteria. *Inclusion criteria* were: diagnosis of psoriasis, planned switch to systemic therapy and/or to intensified local therapy, an adequately long wash out period, sufficient German language skills, and an age between 18 and 75 years. *Exclusion criteria* were: refusal of study participation and the inability to give informed consent (e.g., in case of mental disability or insufficient German language abilities). Measurements were conducted at two time points. T1 took place before the beginning of a new treatment episode, e.g., the switch to systemic therapy and/or intensified local therapy. T2 took place about twelve (to a maximum of sixteen) weeks after T1, between January 2016 and March 2020. Patients who were enrolled at T1 but could not be followed up at T2 were defined as drop outs.

A control group of skin-healthy participants has been recruited at the same time, using personal contacts and advertisements in newspapers and at public places. The healthy participants were also interviewed with the SCID-I and answered the questionnaires, as mentioned below (Section Psychological Assessment), but only at one time point between T1 and T2.

### Dermatological Assessment

At both time points (T1 and T2), a dermatologist, supervised by SA, examined the study participants medically. He or she conducted a detailed somatic and psychosocial anamnesis, confirmed the diagnosis of psoriasis and assessed disease severity. For the latter purpose, the Psoriasis Area and Severity Index (PASI) was applied. Additionally, PSO rated the severity of their skin lesions using the self-administered PASI and subjective BSA.

The *Psoriasis Area and Severity Index* (PASI) (35) assesses the severity of psoriasis including the criteria erythema, induration and desquamation on a 5-point-scale and the skin area affected (for head, arms, legs, trunk). The sum of all three severity parameters is calculated for the respective body sections, multiplied by the area score for that area (0% = 0, <10% = 1, 10–29% = 2, 30–49% = 3, 50–69% = 4, 70–89% = 5, 90–100% = 6) and multiplied by the weight of the respective section (0.1 for head, 0.2 for arms, 0.3 for body/trunk and 0.4 for legs). A single score is calculated (range: 0 “no disease” to 72 “maximal disease”), combining the four subscores. A PASI score > 10 can be evaluated as moderate to severe (36). The PASI was assessed by a dermatologist before and about 12 weeks after the beginning of a new treatment episode.

#### Self-Report Measures of Psoriasis Severity

The *Body Surface Area* (BSA) assesses the proportion (%) of the skin area affected by psoriasis. For this purpose, a silhouette of the front and back of a body was used in order to draw in the areas covered with psoriatic lesions. The area of an adult's hand can be used as mark, corresponding to cover 10% of the head, 5% of the arm, 3.3% of the trunk and 2.5% of the leg. The BSA was subjectively assessed before and about 12 weeks after the beginning of a new treatment episode and checked by a dermatologist. A BSA of ≤ 10 can be regarded as mild, >10% as moderate to severe (36).

The *subjective PASI* (37) was used as structured self-measurement of psoriasis disease severity by the PSO. It was applied both at T1 and T2. The SAPASI consists of a silhouette of the front and back of a body. Patients shade the affected areas and record the redness, thickness, and scaling of an average psoriatic lesion in the four body sections (head, legs, arms, trunk) on three modified visual analog scales (length 120 mm) with five marks, corresponding to none (0), light (1), moderate (2) severe (3) and extraordinarily severe (4). Additionally, an independent investigator (supervised by SA) evaluates the four body sections on a scale with a range from 0 “no disease” to 6 “maximum disease”, depending on the body areas affected by psoriasis. The body sections are differentially weighted (0.1 for head, 0.2 for arms, 0.3 for body/trunk and 0.4 for legs). The following formula was used for the calculation of the SAPASI (38).


$$\begin{array}{l} \text{SAPASI }=\;[\left(0.1\;*\text{ Ah}\right)+\left(0.2\;*\text{ Au}\right)+\left(0.3\;*\text{ At}\right)+\left(0.4\;*\text{ Al}\right)\;*\\ \;\left[\frac{4\;*\;\left(\text{VASe}+\text{VASi}+\text{VASd}\right)}{\text{VASLength}}\right] \end{array}$$

Ah = score head, Au = score upper extremities, At = score trunk, Al = score lower extremities, VAS e/i/d = visual analog scale erythema/induration/desquamation.

A single score was calculated with a range between 0 and 72. The psoriasis was accordingly classified to be moderate with a score between 3 and 15. It shows a highly predictive validity for the investigator's PASI score (38).

#### Skin-Related Quality of Life

The *Dermatology Life Quality Index* (DLQI) (39, 40) was applied as 10-item self-report measurement, both at T1 and T2. The DLQI assesses the impact of the psoriasis and its treatment on the patients' lives (e.g., leisure time, job, school, studies, relationships), during the last seven days. The items were evaluated on a 5-point Likert scale (range no/not at all = 0, very strong = 3), leading to a minimum value of 0 and a maximum of 30. The higher the DLQI score the lower the skin-related quality of life. Internal consistency can be evaluated to be good, at both time points (T1/T2: Cronbach's α = 0.85/0.84).

### Psychological Assessment

Also at T1, participants were assessed for a current or life-time diagnosis of a psychological disorder using the German version of the Structured Clinical Interview for the Diagnostic and Statistical Manual (DSM) IV—SCID-I (41, 42), which identifies for instance affective-, psychotic-, substance abuse-, anxiety-, somatoform-, eating- and adjustment disorders. Questions screen for axis-I-disorders that should then be assessed in more detail by trained examiners. The high extent of structuring and jump rules, guarantee a high degree of objectivity. In the present study, all SCID-interviews were supervised by an experienced psychotherapist (GBW). Current evidence verify a satisfactory to good retest-reliability for the SCID-I (Kappa range 0.61–0.83) (43).

#### Questionnaires

Participants were asked to fill in several self-report questionnaires, in order to assess the currently perceived, acute stress, traumatic life-time stress during childhood/adolescence (chronic stress), anxiety/depression and coping with psoriasis.

#### Assessment of Currently Perceived Stress and Traumatic Life-Time Stress

The *Perceived Stress Scale* (PSS) (44) is a 14-item self-report measurement and was used to assess the extent of stress load (e.g., uncontrollable, unpredictable, potentially overwhelming situations) during the last month, before (T1) and after the beginning of a new treatment episode (T2). The items were evaluated on a 4-point scale (with a range from 0 = “almost never” to 4 “very often”). A sum score was calculated, leading to a range between 0 and 56. The internal consistency Cronbach's α was unsatisfactory in the present study (T1/T2: Cronbach's α = 0.54/0.55).

The perceived association between the occurrence of a stressful life-event and the onset/exacerbation of psoriasis was assessed using two simple yes/no-questions.

In order to gain insight into the extent of traumatic experiences during childhood, the *Childhood Trauma Questionnaire* (CTQ) (45) was used at recruitment/before the beginning of a new treatment episode (T1). The questionnaire consists of 31 items assessing experiences of abuse (emotional, sexual, physical) and neglect (emotional, physical) during one's childhood and adolescence. The items were rated on a 5-point Likert-scale (0 = “not at all”, 4 = “very often”). A simple sum score was calculated out of 25 items, leaving out the items 10, 16, 22, 29, 30, 31. The sum score had a range between 0 (no abuse/neglect) and 20 (extreme experience of abuse/neglect). Besides the subscale physical neglect (0.45), overall Cronbach's α can be evaluated to be excellent in the present study (0.91, range 0.83 for emotional abuse to 0.94 for sexual abuse).

#### Assessment of Anxiety/Depression

The *Hospital Anxiety and Depression Scale* (HADS) (46, 47) was applied at two time points, before (T1) and after (T2) the beginning of a new treatment episode. It is a 14-item self-report measurement, applied to assess symptoms of anxiety and depression in somatically ill patients during the last week. Physical symptoms (e.g., headache, weight loss) were left out, in order to diminish the rate of false positive judgments. Items were rated on a 4-point Likert scale (0 = “strongly disagree”, 3 = “strongly agree”). A simple sum score was calculated for each of the two subscales (anxiety/depression), with a range between 0 and 21. A cut-off score of ∑ = 11 can be regarded as suitable, leading to an appropriate sensitivity and specifity for the assessment of clinically relevant depressive and anxiety symptoms (47). The HADS turned out in different studies to be a change sensitive measurement (48). In the present study, the reliability can be regarded to be good (T1/T2: Cronbach's α = 0.88/0.91).

#### Assessment of Coping

Coping with psoriasis was assessed using the *Marburg Skin questionnaire* (Marburger Haut-Fragebogen, MHF) at T1 and T2 (49). It consists of 51 items covering coping with psoriasis in different areas (social fears, helplessness, itching-scratching cycle, depression/anxiety, search for information, quality of life). The nine items of the subscale *itching-scratching* were rated on a five-point Likert scale (1 = “not at all” to 5 = “very strong”). A simple sum score was calculated for each of the six subscales (total range 51–254). In the present study, only the intensity of itching/scratching at T1 was of interest, since bi-directional correlations with the psoriasis severity (SAPASI, BSA, PASI, DLQI), delta scores and psychological factors (HADS anxiety/depression, CTQ subscales, PSS “*perceived stress*”) were supposed. Higher values on itching-scratching mean a higher extent of the respective dimension of interest. The internal consistency was satisfactory with Cronbach's α = 0.78 (T1).

### Statistical Analyses

Whether values were missing completely at random (MCAR) or not was analyzed using MCAR tests. It could be proven that all missing values were missing completely at random (all *p*-values > 0.05). Following, analyses were realized using incomplete cases. Nevertheless, in order to check the robustness of our study results, we reanalyzed data using multiply imputated data, taking into account SAPASI at T1, PSS at T1, SAPASI at T1, Delta SAPASI for multiple imputation with four iterations (Supplementary Tables S5, S6).

Continuous and metric variables were controlled for normal distribution, using the Kolmogorov Smirnov test. In case of non-normally distributed or ordinal data, Mann-Whitney U tests, Wilcoxon signed rank tests or Spearman's correlation analyses were applied. Values of the childhood trauma questionnaire showed a right-skewed distribution. Therefore, we used an inverse transformation of the CTQ total value. We decided to use the CTQ total score for further analyses, because of the differing reliabilities of the CTQ subscales, caused in particular by the low internal consistency of the CTQ subscale physical neglect.

Normally distributed, metric variables were compared, using the *t*-test (for independent samples). Homoscedasticity as prerequisite for the *t*-test (for independent samples) was controlled for using the Levene test. Categorical variables were compared using the Chi-squared test. Wilcoxon signed rank tests were applied, in order to assess the change of psoriasis severity (PASI, SAPASI, BSA), anxiety/depression and health-related quality of life (DLQI) between T1 and T2.

Delta scores (difference scores between T2-T1) were calculated for the dependent variables (SAPASI, DLQI), subjected to correlation analyses (Spearman's rank correlations) with CTQ, PSS, HADS, age, gender, among others, and integrated as dependent variables in moderator analyses. In order to test for moderator effects of the perceived stress and childhood trauma, PSS at T1 and the CTQ total score were included as moderator variables, using the Macro Modell Process, version 4.0, by Hayes (50) (documentation available at www.guilford.com/p/hayes3). A heteroscedasticity consistent standard error and covariance matrix estimator as well as bootstrap samples (*n* = 5,000) were applied. The bootstrapping approach allows moderator analyses, despite the low sample size (51). In order to replicate the results from the moderator analyses, hierarchical regression analyses were applied, additionally. With respect to the primary outcome variables Delta DLQI and Delta SAPASI, two models were tested. Model 1 included Delta SAPASI, age, gender as regressors in the first step, *perceived stress* (PSS) at T1 in a second and the interaction term Delta SAPASI × PSS in a third step. Model 2 included Delta SAPASI, age, gender as regressors in a first step, CTQ *total* in a second and the interaction term Delta SAPASI × CTQ *total* in a third step.

Results from Spearman's correlations between subjective disease severity (SAPSAI)/skin-related quality of life and itching/scratching at T1 (as assessed by the questionnaire MHF) were only of minor interest. Above, itching/scratching was significantly correlated with the CTQ subscales/total score and perceived stress (PSS). In order to avoid the effect of multicollinearity, we did not consider the MHF-subscale itching/scratching in the further moderator analyses. Above, since the diagnosis of psoriasis arthritis showed no point-biserial correlations with measures of psoriasis severity, delta values of psoriasis severity/skin-related quality of life and psychological variables (all *p*-values ≥ 0.167), it was not included in the further analyses.

Significant interaction terms between CTQ total × baseline values of psoriasis severity/DLQI were further examined using a median split to form separate subgroups of PSO with mild (≤ 50. percentile) vs. severe psoriasis (>50. percentile). Likewise, groups with a remission of psoriasis severity to 75% from the SAPASI value at T1 (75% responder vs. non-responder), were subjected to Spearman's correlation analyses between CTQ total and Delta SAPASI/Delta DLQI.

We did not consider the course of the PASI scores, because of a large amount of missing values. This was owed to the fact that after PSO had been successfully diagnosed and a new treatment episode started, they were discharged from our Department of Dermatology to an outpatient unit close to their home. Thus, the PASI could not be obtained on the second time point in many cases.

For all analyses, z-standardized values were taken into account. In order to control for multiple testing, Bonferroni-corrected *p*-values were applied. Results were considered significant at a significance level of *p* ≤ 0.05/number of statistical tests forming a family. *P*-values ≤ 0.001 were considered as highly significant. The above mentioned statistical analyses were mainly realized using SPSS 28.0.0.0.

## Results

### Drop Out Analysis

PSO included in the present study did not significantly differ from dropped out PSO, with respect to the sociodemographic and clinical characteristics. However, dropped out PSO showed higher values on the subscale HADS anxiety, and a higher impairment of the skin-related quality of life (DLQI) (see Supplementary Table S1).

### Sociodemographics

Most PSO were enrolled during a stay on the inpatient ward (*n* = 56, 67.5%). Two third (62.7%) of the PSO were male and 50% between 37.8 and 62.5 years old (range: 20–75 yrs). About every second was married and two third had 10 years of school education. PSO had a significantly lower educational level and were more often unable to work than controls (see Table 1).

**Table 1:** Characteristics of the sample of patients with psoriasis (PSO) and skin-healthy control group (HC).

	**PSO *n* (%) median (IQR)**	**HC** *n* **(%)** **median (IQR)**	**t/H/U/χ^2^**	***p***
Total number of participants	**83 (100)**	**66 (100)**		
Men	52 (62.7)	33 (50.0)	χ^2^ = 2.401	0.121
Women	31 (37.3)	33 (50.0)		
Age in years	53.7 (37.8–62.5)	51.5 (33.3–59.2)	U = 2,507.500	0.376
Min/Max	20.1/74.9	18.6/69.5		
Family status			χ^2^ = 1.559	0.459
Single	15 (18.0)	14 (21.2)		
Married	36 (43.4)	33 (50.0)		
Cohabited	32 (38.6)	19 (28.8)		
Education			χ^2^ = 25.131	**<0.001**
<10 years	13 (15.7)	5 (7.6)		
=10 years	52 (62.7)	20 (30.3)		
>10 years	18 (21.6)	41 (62.1)		
Unability to work	22 (27.8)^a^	1 (1.5)	χ^2^ = 18.683	**<0.001**
Smokers	22 (26.5)	15 (22.7)	χ^2^ = 0.425	0.514
Number of cigarettes/day (mean, SD)	12.3 (5.7)^b^	8.8 (6.0)^b^	*t* = 1.716	**0.048**
Regular alcohol	39 (47.0)	34 (51.5)	χ^2^ = 0.302	0.583
Regular sport	22 (26.5)	43 (65.2)	χ^2^ = 22.326	**<0.001**
Body mass index, BMI in kg/m^2^, min/max	27.1 (24.1–31.3)^b^ 17.0/53.9	24.2 (22.1–27.2)^b^ 18.6/25.2	F = 14.228	**<0.001**
Allergies (e.g., house dust, food)	23 (28.4)^c^	21 (31.8)	χ^2^ = 0.203	0.652
Autoimmune diseases	9 (11.1)^d^	5 (7.6)	χ^2^ = 0.528	0.468
Infectious diseases	4 (4.8)	3 (4.5)	χ^2^ = 0.006	0.937
Thyroid diseases	10 (12.0)	7 (10.6)	χ^2^ = 0.076	0.783
Heart diseases	14 (16.9)	7 (10.6)	χ^2^ = 1.190	0.275
Circulatory diseases (e.g., hypertension)	37 (44.6)	15 (22.7)	χ^2^ = 7.726	**0.005**
Lung diseases	7 (8.5)^e^	7 (10.6)	χ^2^ = 0.183	0.669
Liver diseases	17 (20.5)	2 (3.0)	χ^2^ = 10.064	**0.002**
Kidney diseases	3 (3.6)	1 (1.5)^f^	χ^2^ = 0.597	0.440
Digestive tract diseases	3 (3.6)	2 (3.0)	χ^2^ = 0.5039	0.844
Metabolic diseases	15 (18.3)^g^	6 (9.1)	χ^2^ = 2.543	0.111
Musculoskeletal diseases	7 (8.4)	5 (7.6)	χ^2^ = 0.037	0.848
Blood diseases	5 (6.0)	1 (1.5)	χ^2^ = 1.934	0.164

a *4 missing values*.

b *PSO: 1 missing value, HC: 3 missing values*.

c *2 missing values*.

d *2 missing values*.

e *1 missing value*.

f *1 missing value*.

g *1 missing value*.

*Categorical data are given as n and percentage of total number, continuous data as median and Interquartile Range (IQR) or mean and Standard Deviation (SD). Bold values indicate significance at p ≤ 0.05*.

### Medical Characterization

PSO showed a significantly higher body mass index (BMI), smoked more cigarettes per day and did less sport compared to controls (Table 1). Also, significantly more PSO were unable to work, presented cardiovascular diseases (e.g., hypertension) or liver diseases (e.g., hepatitis) (see Table 1).

The majority of the patients (*n* = 70, 84.3%) had a pure diagnosis of psoriasis vulgaris (ICD-10: L40.0), while the rest suffered from other forms of psoriasis (L40.3 palmoplantaris *n* = 6–7.2%, L40.0/L40.3 vulgaris et palmoplantaris *n* = 4–4.8%, L40.0/L40.4 Psoriasis vulgaris et guttata *n* = 2–2.4%, L40.0/L40.1 Psoriasis vulgaris et pustulosa et generalista *n* = 1–1.2%). *n* = 23 patients (27.7%) also had a diagnosis of psoriasis arthritis (ICD-10: L40.52), additionally to a diagnosis of L40.0-4. The median PASI (13.8, IQR 10.4, 20.1) corresponded to a medium to severe form of psoriasis. Likewise, the median of the affected body surface area (BSA) was 18.0 (IQR 9.0, 40.0) and of the SAPASI 15.6 (IQR 7.4, 24.2), which indicated a medium to severe manifestation of psoriasis. According to the PASI cut-off score > 10, *n* = 59 (71.1%) and to the BSA cut-off score > 10, *n* = 54 (67.5%) presented a moderate to severe form of psoriasis. Based on the SAPASI cut-off score > 15, *n* = 44 (53.0%) of the PSO showed a severe manifestation of psoriasis. A median of eight areas were affected (IQR 5.8, 10.0). Body areas most often affected were: the legs (*n* = 69, 84.1%), bottom (*n* = 63, 75.9), arms (*n* = 62, 75.6%), trunk (*n* = 61, 74.3), feet (*n* = 49, 59.8%), hands (*n* = 49, 59.8%), head (*n* = 46, 56.1%) and genital area (*n* = 31, 37.8%). In nearly every second patient (*n* = 38/45.8%) also finger nails were affected. Median age at onset of the psoriasis was 30.1 years (IQR 17.4, 41.4). Median duration was 18.0 years (IQR 6.7, 27.2).

Of the included patients, *n* = 34 (41.0%) received a conventional systemic therapy (e.g., with Methotrexat, Cyclosporin, Acitretin, Apremilast) or a combination of conventional systemic therapy and intensified local therapy. *n* = 56 patients (67.5%) began a new therapy with a biologic (most of them with the IL-17A-inhibitor secukinumab: *n* = 29/34.9%, others: TNF-α inhibitor, IL-12/23 inhibitor, IL-23 inhibitor: *n* = 19/22.9%) or received a combination of biologic and intensified local therapy (*n* = 8/9.6%). *n* = 10 patients (12.0%) received intensified local therapy only, phototherapy or a combination of both (see Supplementary Figure S1).

Four patients received a psychiatric comedication during the new treatment episode (one phenothiazine, one serotonin-norepinephrine reuptake inhibitor, one gabapentinoid, one selective serotonin reuptake inhibitor).

### Mental Health Assessment at Baseline (T1) and T2

The SCID revealed that *n* = 30 (37.3%) of the PSO showed a current or life-time diagnosis of a mental disorder. Of them, *n* = 18 (21.7%) had only one SCID diagnosis, *n* = 12 (14.4%) had two or more. Affective disorders (F3X.X, *n* = 24/28.9%) and anxiety disorders (F40.X, F41.X, F42.X, F43.X, *n* = 24/28.9%) were most often coded (see Supplementary Figure S2).

#### Assessment of Childhood Trauma

The Childhood Trauma Questionnaire (CTQ) identified significantly more PSO that reported frequent physical neglect (significant at Bonferroni-corrected *p*-value ≤ 0.01) than skin-healthy controls. Other CTQ subscales did not significantly differ between groups (Table 2).

**Table 2:** The table shows differences between patients with psoriasis (PSO, *n* = 83) and healthy controls (*n* = 66) with respect to childhood trauma (CTQ), perceived stress (PSS) and anxiety/depression (HADS).

	**T1**				**T2**					
	**Median (IQR)**		**U**	***p***	**Median (IQR)**		**U**	***p***	**Z (Delta T1-T2)**	***p*** **(Delta T1-T2)**
	**PSO (*n* = 83)**	**HC (*n* = 66)**			**PSO (*n* = 83)**	**HC (*n* = 66)^g^**				
CTQ total	15.5 (12.0–23.8)^a^	14.0 (11.0–21.3)	2,113.000	0.262^#^						
CTQ-emotional abuse	0.0 (0.0–3.0)^b^	1.0 (0.0–3.0)^d^	2,138.500	0.640^#^						
CTQ-physical abuse	0.0 (0.0–1.0)^c^	0.0 (0.0–0.0)	2,153.000	0.156^#^						
CTQ-sexual abuse	0.0 (0.0–0.0)^b^	0.0 (0.0–0.0)	2,161.000	0.123^#^						
CTQ-emotional neglect	4.0 (1.0–7.5)^c^	2.0 (0.0–5.3)	1,862.500	0.020^#^						
CTQ-physical neglect	1.0 (0.0–3.5)^c^	0.0 (0.0–1.0)	1,818.500	**0.007** ^#^						
PSS	30.0 (25.0–34.0)^e^	26.0 (21.0–30.0)^f^	1,404.000	**0.005** ^#^	27.0 (21.3–32.0)^d^	26.0 (21.0–30.0)^f^	2,230.500	0.383	−2.279	0.023^#^
HADS-total score	10.0 (5.0–15.0)	5.0 (3.0–9.0)	1,746.500	**<0.001** ^~^	10.0 (5.0–14.0)	5.0 (3.0–9.0)	1,867.500	**<0.001** ^~^	−0.467	0.641^#^
HADS-anxiety	5.0 (2.0–8.0)	3.0 (2.0–6.0)	2,182.500	0.032^~^	6.0 (3.0–8.0)	3.0 (2.0–6.0)	1,957.500	**0.003** ^~^	−0.889	0.374^#^
HADS-depression	5.0 (2.0–8.0)	2.0 (1.0–3.3)	1,578.500	**<0.001** ^~^	4.0 (1.0–7.0)	2.0 (1.0–3.3)	1,868.500	**<0.001** ^~^	−1.868	0.062^#^
DLQI	7.0 (3.0–13.0)				2.0 (0.0–5.0)				−6.229	**<0.001** ^#^
SAPASI^h^	15.6 (7.4–24.2)				2.4 (0.8–6.6)				−6.097	**<0.001** ^#^
BSA^d^	18.0 (9.0–40.0)				5.0 (2.0–10.0)				−6.480	**<0.001** ^#^
PASI^i^	13.8 (10.4–20.1)				3.3 (1.4–7.1)				−5.972	**<0.001** ^#^

a *11 missing values*.

b *12 missing values*.

c *10 missing values*.

d *3 missing values*.

e *18 missing values*.

f *5 missing values*.

g *Numbers are colored in gray to mark that values are the same as at T1 since the HCs were only asked to answer PSS and HADS once (at T1)*.

h *14 missing values*.

i *57 missing values*.

# *Significance at Bonferroni-corrected p-value ≤ 0.01*.

~ *Significance at Bonferroni-corrected p-value ≤ 0.02*.

*BSA, Body Surface Area; CTQ, Childhood Trauma Questionnaire; DLQI, Dermatology Life Quality Index; HADS, Hospital Anxiety and Depression Scale; PASI, Psoriasis Area and Severity Index; PSS, Perceived Stress Scale; SAPASI, Self-administered Psoriasis Area and Severity Index; T1, before beginning of a new treatment episode; T2, 3 months after the beginning of a new treatment episode*.

*The differences between T1 and T2 with respect to the patient-reported outcomes (BSA, HADS, PSS, SAPASI) and clinical outcome (PASI) are displayed in PSO only. For the outcome parameters the medians and Interquartile Ranges (IQR) are reported. Bold values indicate significance at Bonferroni-corrected p-values*.

#### Perception of Acute Stress in Patients and Controls

At T1, PSO presented a significantly higher level of stress, as measured with the Perceived Stress Scale (PSS), compared with skin-healthy controls (median: 30.0 vs. 26.0, *p* = 0.005). At T2, the PSS did no longer differ between groups (median: 27.0 vs. 26.0, *p* = 0.383) (see Table 2).

*n* = 31 (37.3%) of the PSO reported a first manifestation of the psoriasis after a stressful life-event and *n* = 49 (59.0%) reported an exacerbation of the psoriasis under psychosocial stress.

#### Depression and Anxiety Scores in Patients and Controls

Compared with HC, PSO showed a significantly higher HADS total score and depression subscore at T1. At T2, PSO also presented a significantly higher anxiety subscore than controls (see Table 2). Also, significantly more PSO were above the HADS cut-off score (∑≥11), at both T1 and at T2 (T1: PSO vs. controls: *n* = 41/49.4% vs. *n* = 12/18.2%; χ^2^ = 15.632, df = 1, *p* < 0.001; T2: psoriasis vs. controls: *n* = 36/43.4% vs. *n* = 12/18.2; χ^2^ = 10.685, df = 1, *p* < 0.001). The rate of identified cases in PSO did not significantly change between T1 and T2 (McNemar Test: χ^2^ = 0.552, *p* = 0.458).

### Course of Clinical and Self-Report Outcomes (PRO) Between T1 and T2

Overall, PSO showed a significant improvement from T1 to T2 with respect to the PASI score (Z = −5.972, *p* < 0.001), as well as in the key dermatological self-report parameters SAPASI (Z = −6.097, *p* = < 0.001), BSA (Z = −6.480, *p* < 0.001) and DLQI (Z = −6.229, *p* < 0.001) (see Table 2; Supplementary Figure S3a). Hence, PSO improved with respect to (self-rated) disease severity and quality of life. *n* = 41 (49.4%) of the PSO could be classified as responders, showing a remission of psoriasis severity of 75% (or higher) from the SAPASI value at T1. Above, the perceived stress also decreased over time (Z = −2.279, *p* = 0.023). However, the HADS subscales did not significantly decrease in response to the start of a new dermatological treatment scheme (Supplementary Figure S3b).

### Inter-correlations Between Childhood Trauma, Psoriasis Severity Outcomes (PASI, SAPASI, BSA) and Psychological Outcomes (Anxiety/Depression, PSS, DLQI) of the Dermatological Treatment

CTQ was not associated with measures of psoriasis severity (PASI, SAPASI, BSA), neither at T1 nor at T2 (see Supplementary Table S2). We found significantly positive correlations between CTQ subscales physical abuse, emotional neglect and HADS- anxiety/depression, both at T1 and T2; for *emotional abuse* only at T1 (applying a Bonferroni-corrected *p*-value of *p* ≤ 0.01) (*emotional abuse* × HADS T1/T2, p T1/p T2: Spearman's rho = 0.308/0.247, *p* = 0.009/0.038; *physical abuse*: 0.431/0.391, *p* < 0.001/ < 0.001; *sexual abuse*: 0.241/0.154, *p* = 0.043/0.199; *emotional neglect*: 0.414/0.356, *p* < 0.001/0.002; *physical neglect*: 0.203/0.066, *p* = 0.085/0.578). A higher extent of traumatic childhood experiences (in particular *physical abuse or emotional neglect*) was hence associated with higher anxiety/depression at both measurement points.

CTQ subscales physical abuse and emotional neglect showed significant correlations with the PSS, both at T1 and T2; for *emotional/sexual abuse* only at T1 (applying a Bonferroni-corrected *p*-value of *p* ≤ 0.01) (CTQ subscales × PSS, Spearman's rho T1/T2, pT1/pT2, *emotional abuse*: 0.383/0.242, *p* = 0.002/0.047, *physical abuse*: 0.557/0.435, *p* < 0.001/ < 0.001, *sexual abuse*: 0.352/0.167, *p* = 0.004/0.174, *emotional neglect*: 0.547/0.342, *p* < 0.001/0.004, *physical neglect*: 0.313/0.057, *p* = 0.011/0.639). Patients with a higher extent of traumatic childhood experience (in particular *physical abuse or emotional neglect*) also reported higher perceived stress. At T2, no correlation could be shown with the CTQ subscales *emotional abuse, sexual abuse* and *physical neglect*.

We found a significant correlation between the CTQ subscale physical abuse and the DLQI at T1 (applying a Bonferroni-corrected *p*-value of *p* ≤ 0.01) (Spearman's rho T1/pT1, *emotional abuse*: 0.205/0.087, *physical abuse*: 0.310/0.008, *sexual abuse*: 0.272/0.022, *emotional neglect*: 0.257/0.028, *physical neglect*: 0.161/0.174). The higher the CTQ subscale score *physical abuse*, the lower the skin-related quality of life (higher DLQI values). At T2, no associations turned out to be significant.

### Inter-correlations Between Childhood Trauma/Perceived Stress, Coping, Anxiety/Depression With Therapy Outcomes (Delta SAPASI, Delta DLQI)

The CTQ subscale physical abuse was significantly correlated with improvement of the skin-related quality of life (Delta DLQI) (applying a Bonferroni-corrected *p*-value of *p* ≤ 0.01). Increased values on the CTQ-subscale *physical abuse* were associated with a better dermatological outcome (higher decrease of Delta DLQI, Spearman's rho = −0.376, *p* = 0.001) (see Table 3). This correlation could be proven in the group of 75% responders, but not non-responders (Bonferroni-corrected *p*-value of *p* ≤ 0.03, responders: Spearman's rho = −0.503, *p* = 0.003; non-responders: Spearman's rho = −0.403, *p* = 0.041) (see Table 3).

**Table 3:** Correlation analyses between change scores of psoriasis severity (Delta SAPASI), skin-related quality of life (Delta DLQI) and childhood trauma (CTQ, subscales), itching/scratching (MHF), anxiety/depression (HADS), perceived stress (PSS), age, gender and psoriasis arthritis.

**Spearman's rho (*p*-values)**	**Delta SAPASI**	**Delta DLQI**
CTQ total	−0.239 (0.071)^a^^#^	−0.203 (0.087)^b^^#^
CTQ emotional abuse	−0.291 (0.028)^b^^#^	−0.225 (0.059)^h^^#^
CTQ physical abuse	−0.278 (0.033)^c^^#^	–**0.376 (0.001)**^i^^#^
CTQ sexual abuse	−0.008 (0.956)^b^^#^	−0.118 (0.329)^h^^#^
CTQ emotional neglect	−0.233 (0.076)^c^^#^	−0.245 (0.037)^i^^#^
CTQ physical neglect	−0.184 (0.162)^c^^#^	−0.181 (0.126)^i^^#^
MHF itching scratching (T1)	–**0.286 (0.017)**^d^^~^	–**0.484 (<0.001)**^j^^~^
MHF itching scratching (T2)	0.034 (0.782)^e^^~^	−0.001 (0.992)^j^^~^
HADS anxiety (T1)	0.059 (0.630)^d^^#^	−0.270 (0.013)^m^^#^
HADS depression (T1)	0.148 (0.224)^d^^#^	–**0.286 (0.009)**^m^^#^
HADS anxiety (T2)	0.106 (0.385)^d^^#^	0.027 (0.809)^m^^#^
HADS depression (T2)	0.137 (0.262)^d^^#^	0.059 (0.599)^m^^#^
PSS (T1)	−0.040 (0.777)^f^^~^	–**0.297 (0.016)**^k^^~^
PSS (T2)	0.090 (0.471)^g^^~^	−0.022 (0.846)^l^^~^
SAPASI (T1)	–**0.710 (<0.001)**^d^^~^	–**0.328 (0.003)**^l^^~^
Delta SAPASI	1	**0.526 (<0.001)** ^d^ ^~^
DLQI (T1)	–**0.317 (0.008)**^d^^°^	–**0.702 (<0.001)**^m^^°^
Age	**0.278 (0.021)** ^d^ ^°^	0.110 (0.324) ^m^^°^
Gender	0.123 (0.312)^d^^°^	0.164 (0.139) ^m^^°^
psoriasis arthritis	−0.095 (0.437)^d^^°^	−0.050 (0.653) ^m^^°^

a *n = 58*.

b *n = 57*.

c *n = 59*.

d *n = 69*.

e *n = 68*.

f *n = 52*.

g *n = 66*.

h *n = 71*.

i *n = 73*.

j *n = 82*.

k *n = 65*.

l *n = 80*.

m *n = 83*.

# *Significance at Bonferroni-corrected p-value ≤ 0.01*.

~ *Significance at Bonferroni-corrected p-value ≤ 0.03*.

° *Significance at p-value ≤ 0.05*.

*BSA, Body Surface Area; DLQI, Dermatology Life Quality Index; HADS, Hospital Anxiety and Depression Scale; MHF, Marburg Skin Questionnaire/Marburger Haut-Fragebogen; PSS, Perceived Stress Scale; SAPASI, Self-administered Psoriasis Area and Severity Index. Bold values indicate significance at Bonferroni-corrected p-values*.

Also, the association of itching/scratching at *T1*, assessed *via* Marburg Skin Questionnaire/MHF) (*p* < 0.001), HADS depression *(T1)* (*p* = 0.009) and perceived stress (PSS) *(T1)* (*p* = 0.016) with Delta DLQI turned out to be significant at Bonferroni-corrected p-levels (see Table 3).

More pronounced itching/scratching at T1 was accompanied by a higher improvement of the skin-related quality of life (Delta DLQI, Spearman's rho = −0.484, *p* < 0.001) and subjective skin severity (Delta SAPASI, Spearman's rho = −0.286, 0.017) (Table 3).

Patients with a higher extent of perceived stress (PSS) at T1 and a higher extent of CTQ childhood trauma experiences also showed more *itching/scratching* (*itching/scratching* × PSS T1, Spearman's rho = 0.294, *p* = 0.017; *itching/scratching* × CTQ total score, Spearman's rho = 0.276, *p* = 0.019) (data not shown in Table 3).

Other variables (childhood trauma/perceived stress, anxiety/depression) were not significantly associated with changes of subjective skin severity (Delta SAPASI) at a Bonferroni-corrected *p*-level.

### Inter-correlations Between Baseline Values, Age, Gender, Psoriasis Arthritis With Therapy Outcomes (Delta SAPASI, Delta DLQI)

A higher skin severity (SAPASI) at T1 was significantly associated with a higher improvement of the skin-related quality of life (Delta DLQI) (SAPASI T1 × Delta DLQI, Spearman's rho = −0.328, *p* = 0.003) (applying a Bonferroni-corrected *p*-value of *p* ≤ 0.03). An improvement of the skin severity (Delta SAPASI) was linked with an improvement of the skin-related quality of life (Delta DLQI) (Delta SAPASI × Delta DLQI, Spearman's rho = 0.526, *p* < 0.001) (significant at Bonferroni-corrected *p*-value ≤ 0.03) (see Table 3).

Likewise, a higher impairment of the skin-related quality of life (DLQI) at T1 was associated with a higher improvement of DLQI (DLQI T1 × Delta DLQI, Spearman's rho = −0.702, *p* < 0.001) (significant at *p* ≤ 0.05).

With respect to improvement of the subjective psoriasis severity, a higher baseline skin severity (SAPASI at T1) and skin- related quality of life (DLQI at T1) were negatively associated with improvement of subjective psoriasis severity (SAPASI T1 × Delta SAPASI, Spearman's rho = −0.710, *p* < 0.001; DLQI T1 × Delta SAPASI, Spearman's rho = −0.317, *p* = 0.008). The impact of age on the therapy outcome (Delta SAPASI) turned out to be significant (age × Delta SAPASI, Spearman's rho = 0.278, *p* = 0.021) (Table 3). Older patients benefited from the dermatological treatment to a lesser extent than younger patients. Gender and psoriasis arthritis consistently showed no association, with neither the Delta DLQI nor the Delta SAPASI.

### Determinants of Therapy Outcome (Delta DLQI, Delta SAPASI) in PSO

#### Determinants of the Delta DLQI: Impact of Perceived Stress

With respect to the improvement of DLQI after the beginning of a new treatment episode, two moderator models were tested using the Macro Modell Process (50). Model 1 included perceived stress as moderator, model 2 the CTQ total score. In the first model, an improvement of the subjective psoriasis severity (Delta SAPASI) was positively linked with an improvement of the skin-related quality of life (Delta DLQI) (β = 0.514, *p* < 0.001, 95% CI 0.269–0.759). A higher *perceived stress* at T1 was associated with a higher decrease of DLQI (i.e., an improvement of DLQI) under the dermatological treatment (β = −0.434, *p* < 0.001, 95% CI −0.659, −0.209). An improvement of skin severity from T1 to T2 (negative Delta SAPASI) was associated with a higher improvement of skin-related quality of life (negative Delta DLQI), in particular in patients with a higher experience of perceived stress (higher PSS values at T1) (β = 0.297 *p* = 0.025, 95% CI 0.039–0.555, PSS *perceived stress* at T1 ≤ median: Spearman's rho correlation Delta SAPASI × Delta DLQI = 0.386, *p* = 0.029; PSS perceived stress at T1 > median: Spearman's rho correlation Delta SAPASI × Delta DLQI = 0.824, *p* < 0.001) (applying a Bonferroni-corrected *p*-value of *p* ≤ 0.03). The model explained 47.9% of the total variance [*F*_(5, 46)_ = 8.466, *p* < 0.001] (see Table 4; Supplementary Table S3a; Supplementary Figure S4). The interaction term Delta SAPASI × PSS *perceived stress* additionally explained 6.1% of the data variance (significance of change in *F*: *p* = 0.025) (Supplementary Table S3a).

**Table 4:** Results of the moderator analysis according to the Macro Modell Process by Hayes.

	**Beta**	***t***	**Lower 95% CI**	**Upper 95% CI**	***p***		**Beta**	***t***	**Lower 95% CI**	**Upper 95% CI**	***p***
**Delta DLQI**											
Age	−0.023	−0.197	−0.255	0.209	0.845	Age	−0.037	−0.290	−0.289	0.216	0.773
Gender	0.101	0.430	−0.372	0.573	0.669	Gender	0.128	0.478	−0.408	0.663	0.635
Delta SAPASI	0.514	4.220	0.269	0.759	**<0.001**	Delta SAPASI	0.363	2.687	0.092	0.633	**0.010**
PSS “perceived stress” (T1)	−0.434	−3.886	−0.659	−0.209	**<0.001**	CTQ total (T1)	−0.255	−1.912	−0.523	0.013	0.061
Delta SAPASI × PSS “perceived stress” (T1)	0.297	2.318	0.039	0.555	**0.025**	Delta SAPASI × CTQ total (T1)	0.039	0.368	−0.175	0.253	0.714
*n* = 52, *F*_(5, 46)_ = 8.466, *p* < 0.001, R^2^ = 47.9						*n* = 58, *F*_(5, 52)_ = 3.233, *p* = 0.013, R^2^ = 23.7					

*CTQ, Childhood Trauma Questionnaire; DLQI, Dermatology Life Quality Index; SAPASI, Self-administered Psoriasis Area and Severity Index*.

*Delta SAPASI was included as independent variable. Treatment outcome (Delta DLQI) as dependent variable. Age and gender, were included as covariates, PSS “perceived stress” at T1 and CTQ total as moderators. Bold values indicate significance at p ≤ 0.05*.

#### Determinants of the Delta DLQI: Impact of Childhood Trauma

Taking into account the CTQ total score in a second model, no significant impact could be shown with respect to DLQI (β = −0.255, *p* = 0.061, 95% CI −0.523, 0.013) (Table 4). The model explained 23.7% of the total variance [*F*_(5, 52)_ = 3.233, *p* = 0.013] (see Table 4; Supplementary Table S3b). The interaction term Delta SAPASI × CTQ total score did not explain additional data variance (significance of change in *F*: *p* = 0.714) (Supplementary Table S3b).

The results of the moderator analyses (Model 1 and 2), using the Macro Modell Process, could be largely confirmed by hierarchical regression analyses (see Supplementary Tables S3a,b).

#### Determinants of the Delta SAPASI: Impact of Perceived Stress

**Figure 1:**
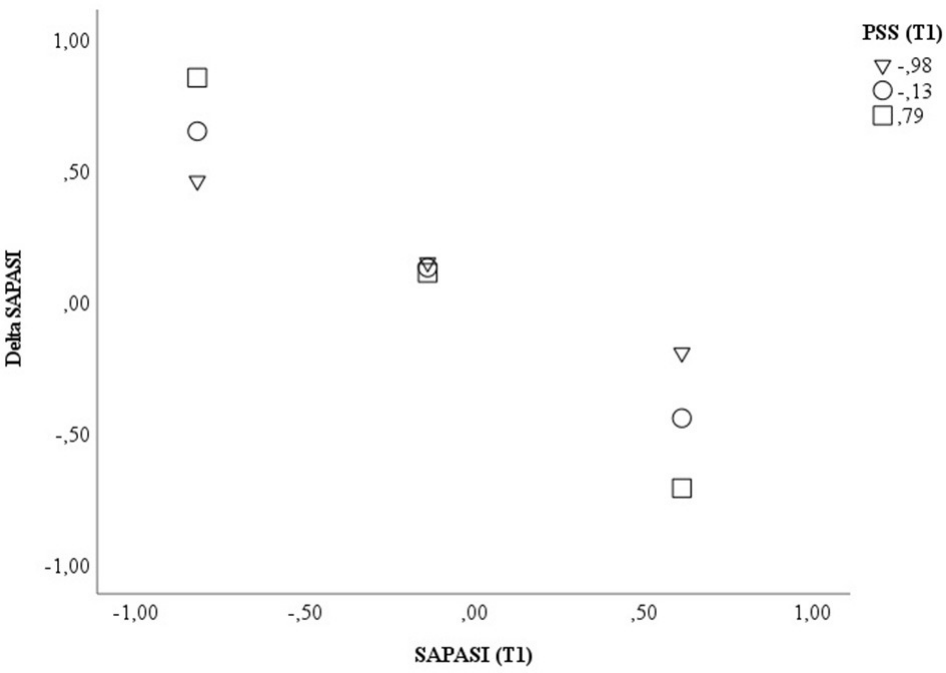
Displayed are the data for visualizing the conditional effect of the predictor SAPASI at T1 on Delta SAPASI, depending on the severity of PSS *perceived stress* at T1. Z-standardized values were used. PSS, Perceived Stress Scale; SAPASI, Self-administered Psoriasis Area and Severity Index.

With respect to the psoriasis severity, we could find a significant impact of the SAPASI at T1 on the treatment outcome (Delta SAPASI) (β = −0.810, *p* < 0.001, 95% CI −1.057, −0.563), after beginning of a new treatment episode. The more severe the psoriasis at T1, the more the psoriatic skin improved after switching to a new therapy. Furthermore, we could show a significant interaction effect between SAPASI at T1 and the *perceived stress* at T1 on psoriasis severity (β = −0.358, *p* = 0.026, 95% CI −0.671, −0.045). In both PSO with a higher or lower extent of *perceived stress*, a higher psoriasis severity at T1 was associated with a higher improvement of psoriatic skin lesions. However, the latter association was more pronounced in the group of PSO with higher perceived stress (see Figure 1). The model explained 54.9% of the total variance [*F*_(5, 46)_ = 11.176, *p* < 0.001] (see Table 5; Supplementary Table S4a). An additional variance of 5.2% could be explained, when the interaction term SAPASI T1 × PSS T1 was included in the final model (significance of change in *F*: *p* = 0.026) (Supplementary Table S4a).

**Table 5:** Results of the moderator analysis according to the Macro Modell Process by Hayes.

	**Beta**	***t***	**Lower 95% CI**	**Upper 95% CI**	***p***		**Beta**	***t***	**Lower 95% CI**	**Upper 95% CI**	***p***
**Delta SAPASI**											
Age	0.196	2.002	−0.001	0.393	0.051	Age	0.090	1.087	−0.076	0.257	0.282
Gender	−0.170	−0.836	−0.578	0.239	0.407	Gender	−0.138	−0.783	−0.490	0.215	0.438
SAPASI (T1)	−0.810	−6.589	−1.057	−0.563	**<0.001**	SAPASI (T1)	−0.728	−7.814	−0.915	−0.541	**<0.001**
PSS “perceived stress” (T1)	−0.070	−0.725	−0.266	0.125	0.472	CTQ total (T1)	−0.206	−2.406	−0.377	−0.034	**0.020**
SAPASI T1 × PSS “perceived stress” (T1)	−0.358	−2.303	−0.671	−0.045	**0.026**	SAPASI T1 × CTQ total (T1)	−0.279	−2.775	−0.480	−0.077	**0.008**
*n* = 52, *F*_(5, 46)_ = 11.176, *p* < 0.001, R^2^ = 54.9						*n* = 58, *F*_(5, 52)_ = 18.635, *p* < 0.001, R^2^ = 64.2					

*CTQ, Childhood Trauma Questionnaire; SAPASI, Self-administered Psoriasis Area and Severity Index*.

*SAPASI at T1 was included as independent variable. Treatment outcome (Delta SAPASI) as dependent variable. Age and gender were included as covariates, PSS ‘perceived stress' at T1 and CTQ total as moderators. Bold values indicate significance at p ≤ 0.05*.

#### Determinants of the Delta SAPASI: Impact of Childhood Trauma

**Figure 2:**
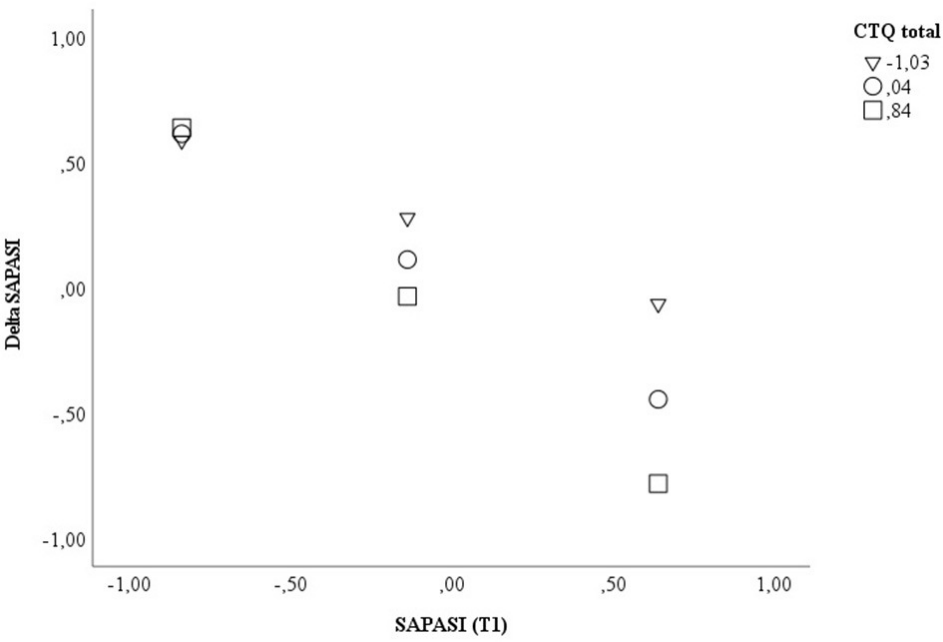
Displayed are the data for visualizing the conditional effect of the predictor SAPASI at T1 on Delta SAPASI, depending on the severity of the CTQ total at T1. Z-standardized values were used. CTQ, Childhood Trauma Questionnaire; SAPASI, Self-administered Psoriasis Area and Severity Index.

In a second model, including the CTQ total score, a significant direct effect (β = −0.206, *p* = 0.020, 95% CI −0.377, −0.034) and interaction effect with SAPASI at T1 could be shown (β = −0.279, *p* = 0.008, 95% CI −0.480, −0.077). In PSO with a higher extent of childhood trauma experiences, the association between psoriasis severity at T1 and improvement of psoriatic skin lesions was higher (CTQ total ≤ median: Spearman's rho correlation SAPASI at T1 × Delta SAPASI = −0.424, *p* = 0.020; CTQ total > median: Spearman's rho correlation SAPASI at T1 × Delta SAPASI = −0.885, *p* < 0.001) (applying a Bonferroni-corrected *p*-value of *p* ≤ 0.03). The model including the CTQ total score explained 64.2% of the total variance [*F*_(5, 52)_ = 18.635, *p* < 0.001] (see Table 5; Figure 2; Supplementary Table S4b for further information).

The results of the moderator analyses (Model 1 and 2), using the Macro Modell Process, could be largely replicated by hierarchical regression analyses (see Supplementary Tables S4a,b). Including the interaction term SAPASI T1 × CTQ total, additionally explained 5.3% of data variance (significance of change in *F*: *p* = 0.008).

## Discussion

### General Discussion

The primary aim of the present study was the examination of acute current stress and the impact of adverse childhood experiences, their interaction and additional psychological symptomatology, on the dermatological therapy outcome in PSO. The results may give hints toward psychological moderators of the therapy outcome in PSO, which should be carefully taken into consideration when the dermatological treatment is planned in order to guide the treatment decision.

We could show a significant improvement of the psoriatic skin lesions under the beginning of a new treatment episode, mainly the beginning of systemic therapy. This was accompanied by a significant improvement of the skin-related quality of life which has been already shown in former studies [for a systematic review: (52)]. One major finding of our study is that the psoriasis severity at T1 was positively associated with the improvement of skin severity under dermatological treatment, mainly with systemic agents. This finding can be explained by the fact that systemic therapy is highly recommended and effective in the treatment of PSO with moderate to severe forms of the disease or psoriasis arthritis. In the present study sample, nearly three quarter (71.1%) of the PSO included suffered from a moderate to severe intensity of psoriasis. Following, a high responsiveness of these PSO to the offered dermatological treatment e.g., by systemic therapy with secukinumab was expected. This is in line with findings suggesting symptom reduction depending on the baseline disease severity (53) and mirrors good clinical practice using the S3-guidelines for the treatment of psoriasis (35).

Main finding was a significantly higher extent of CTQ *physical neglect* experienced by PSO compared with skin-healthy controls. This is in line with existing evidence confirming a relationship between retrospectively reported childhood experiences and psoriasis (14–16, 54). The severity of psoriasis was not correlated with traumatic experiences which is in line with Simonic et al. (14) but contradicts other findings (55). The latter study also applied the CTQ and could show lower values than in our present sample (especially on the subscale sexual abuse). The differences may be explained by peculiarities in the patient sample (55). The above mentioned study excluded PSO with a history of any psychiatric disorders and only patients from an outpatient clinic were enrolled.

Concerning psychological distress, a major finding of our study was the impact of acute stress and childhood trauma on the treatment outcome—mainly self-rated psoriasis severity. For the first time, we could show that the severity of childhood trauma and the level of acute stress, prior to the start of a new treatment scheme, impacts the improvement of psoriatic skin lesions in response to treatment. In PSO with a higher extent of childhood trauma experiences, a higher psoriasis severity at T1 was associated with a more distinct improvement of psoriatic skin lesions. Acute stress perception at T1 had a similar impact, although it decreased in response to the dermatological treatment.

The results confirm a differential effectiveness of antipsoriatic treatment in a certain subgroup of PSO. Patients vulnerable for psoriasis and with experiences of childhood trauma may have altered patterns of inflammatory markers. This is in line with evidence of lasting epigenetic modifications in many target tissues via stress exposure during distinct life periods, especially early childhood when the epigenome shows heightened plasticity to stress exposure (56). Subgroup analyses for specific types of trauma (sexual, physical or emotional abuse) revealed that traumatic experiences may differentially impact single inflammatory parameters (57). In line, new treatment options for PSO, such as systemic therapy, acting as inhibitors for certain pro-inflammatory cytokines [e.g., secukinumab as interleukin(IL)-17 inhibitor], or biologics, acting as TNF-α inhibitors, are effective treatment options for moderate-to-severe psoriasis forms (35).

The deleterious effects of adverse childhood experiences on health outcomes may be partially attributable to dysregulation of the hypothalamic-pituitary-adrenal (HPA) axis, which coordinates the response to stress, and the consequent fostering of a pro-inflammatory environment (58). Stress signals activate the HPA axis and the sympathetic nervous system. Secretagogues, e.g., cortisol, catecholamines and neuropeptides can alter functions of the immune system and promote possible disease states (59). In line, increased IL-17A expression on Th17 cells and on CD4+ regulatory T cells have been found in multiple trauma patients (60). Above, findings suggest higher levels of TNF-α in patients with experiences of sexual abuse (61).

Furthermore, acute psychosocial stress at T1 was associated with a higher improvement of psoriasis severity after switch to systemic therapy. This finding alludes altered patterns of, e.g., pro-inflammatory cytokines under stress in PSO which seems to be normalized under therapy with systemic agents (e.g., IL-17A-, TNF-α-, IL-12/23-, IL-23-inhibitor). A dysfunctional activity of the HPA axis may be assumed leading to an impaired production of glucocorticoids in situations of adversity in PSO. Thus, the pro-inflammatory effects of stressors may not be counteracted in these patients. In line, rats exposed to a chronic social contact stress paradigm presented psoriatic skin lesions when they were treated with glucocorticoid receptor blockers and showed a down-regulated corticosterone secretion (62). However, existing findings rather indicate no altered HPA axis function in PSO under acute psychosocial stress (10). After all, current evidence remains inconclusive, since changes of endocrinological stress parameters (decreased salivary cortisol, increased epinephrine/norepinephrine) and immune functions (increased monocyte number and number of CD4+cells) could be shown as well (9, 10, 63).

Moreover, one may carefully conclude that PSO who present both childhood trauma (higher CTQ values) and a higher psychosocial stress load (higher PSS values), also show a more pronounced improvement of skin severity. It is conceivable, that traumatic experiences during childhood may lead to exaggerated systemic and intracellular inflammatory responses, e.g., increased gene expression of interleukin-1β and nuclear factor-kB, under stress (64). An increased pro-inflammatory reactivity and diminished habituation under repeated psychosocial stress exposure, may promote the evolvement of psoriasis in vulnerable patients (11). This seems to be independent from the current or life-time diagnosis of a posttraumatic stress disorder since in our present sample, only one patient fulfilled the criteria for a PTSD. Above, current symptoms of anxiety/depression do not seem to have a significant impact on the course of psoriasis severity, however the positive impact of an improved psoriatic skin on the perceived quality of life, may be modulated by perceived psychosocial stress. Nevertheless, psychological comorbidity should be taken into consideration, independently from the psoriasis severity, when the dermatological treatment is planned. In line, in a cross-sectional study, PSO with high subjective distress and low objective measures of psoriasis showed higher depression (65). The authors conclude that PSO with a diminished skin-related quality of life (despite low psoriasis severity) should be also screened for depression. However, the above mentioned study did not assess childhood trauma.

It is also conceivable, that the positive impact of childhood trauma on the effectiveness of the dermatological treatment (mainly of systemic therapy) may be based on the intensity of pruritus and scratching. Patients with a higher extent of traumatic childhood experiences and pronounced perceived stress also showed increased itching/scratching as predominant coping. This is in line with findings reporting more emotional dysregulation and self-harm following interpersonal trauma in childhood (66). Since the dermatological treatments also targeted at reducing pruritus, patients with pronounced itching/scratching benefited the most (67).

Following from the present evidence, patients with previous childhood trauma (e.g., range for CTQ subscale score: 4–10) should receive more highly effective anti-psoriatic treatment (with impact on certain cytocine patterns, e.g., IL-17A-inhibitor) and adjunct or integrated psychological support, e.g., by referral to an outpatient psychotherapist or a consultant specialist in psychosomatic medicine. Aims should be a proper management of itching/scratching and an adequate coping with stress in these patients.

In our sample, more than one third (37.3%) of the PSO had a current or life-time psychological disorder. This is in line with studies using questionnaires for the assessment of clinically relevant psychological symptoms (7, 68). Other studies, using an interview-based assessment of diagnosis even yielded to much higher rates [71%: (69), 90%: (70)]. Dermatologists should take psychological comorbidities and increased psychosocial stress exposure into consideration and offer adjunct psychotherapeutic or psychological support (71). Psychosocial stress may go along with a breakdown of adequate coping strategies, leading to pronounced itching/scratching, increasing anxiety/depression, thus leading to an altered illness perception and dissatisfaction with the antipsoriatic treatment. This has been shown by the fact that a rather low psoriasis severity was associated with higher anxiety/depression, possibly leading to worrying, overwhelming, and additional emotional distress by social withdrawal and avoidance in these PSO [see also (65)].

### Strengths and Limitations of the Study

The present results should be critically evaluated in the context of major strengths and shortcomings of the study. Major strengths are the consecutively enrolled sample of PSO, clearly defined inclusion and exclusion criteria, the longitudinal examination in a naturalistic setting and the assessment of psychological disorders using a structured clinical expert interview. The latter is of importance since most studies in this field do not differentiate between a proper psychological diagnosis (by an expert) and subjectively assessed psychological symptoms (by questionnaires), which may lead to an overestimation of psychological burden in these patients (7).

Limitations were the quitely low sample size of *n* = 83 PSO and even lower sample sizes in the main analyses because of missing data. The low sample size may have led to non-normal data and lower statistical power. Despite this, a *post-hoc* power analysis (using G^*^Power), supposing a medium effect size (f^2^ = 0.15) and seven predictors for multiple linear regression analyses, after all revealed a power of even 68% for the present sample. Nevertheless, a replication of the present findings in a larger sample of PSO is needed. In further studies, sufficiently powered analyses based on linear multiple regressions (fixed model) with R^2^ increase and seven tested predictors necessitate a total sample size of at least 103 patients. However, since the bootstrap approach is supposed to bring about robust estimations, even in small samples, we expect that the significant relationships, already found in a quite small patient sample (with incomplete data), may remain significant when the sample size is increased [e.g., (51)]. In line with this assumption is that we were able to largely replicate our data using multiple imputations for missing data (see Supplementary Tables S5, S6). In the future, sufficiently powered samples are needed, in order to allow patient subgroup analyses (depending on the SCID-based psychological diagnosis or on specific traumatic experiences).

Another limitation is the high number of missing values for the PASI as assessed by a dermatologist, especially at the second measurement point T2. We mainly used the subjective BSA or other subjective measures of the psoriasis severity (SAPASI, DLQI), which are based on self-ratings. However, due to the high inter-correlation, e.g., between SAPASI and PASI we think that a generalizability of our results on the PASI, as objective outcome measure, may be justified.

In our present study, *n* = 53 patients dropped out and differed from the final sample with respect to anxiety and impairment of the health-related quality of life. This may have biased our sample toward less severely impaired patients and may have led to an underestimations of the impact of stress on the treatment outcome. Moreover, the generalizability of our results is narrowed. Nevertheless, an analysis using the last observation caring forward method could largely replicate our results but only at a near-significant level for the impact of childhood trauma on therapy outcome (data on request). Thus, future studies should prevent drop outs by a careful follow-up of patients.

Another limitation concerns the use of the questionnaire CTQ for the assessment of childhood trauma, which is retrospective and thus prone to memory bias. Above, it is conceivable, that some PSO answered in a socially desirable manner which may have led to a lower extent of traumatic childhood experiences in our present sample. Furthermore, one should take into consideration the low reliability for the assessment of perceived stress and the subscale physical neglect which demands caution when interpreting the present results.

Above, a high amount of adverse experiences may occur not only during childhood but during the whole life span. For instance Simonic et al. (15) found a high rate of reported emotional and physical abuse during latency and adolescence in patients with psoriatic arthritis. Future studies should therefore use a more appropriate questionnaire such as the Traumatic Antecedents Questionnaire (TAQ) in order to assess adverse events from early childhood to adulthood.

The present sample of PSO was quite heterogeneous. For instance we did not exclude patients with a palmoplantaris form of psoriasis or included psoriasis guttata. The health-related quality of life and psychological burden may be differently affected in these PSO.

### Conclusions

The present study confirmed higher values of certain childhood trauma experiences in PSO. Above, an impact of childhood trauma and acute psychosocial stress on the therapy outcome could be shown in patients with a moderate to severe psoriasis. Both were associated with a better therapy outcome. The findings may allude that PSO with traumatic experiences during their childhood and acute psychosocial stress before the beginning of a new treatment episode, probably show a distinct pattern of pro-inflammatory cytokines and may thus benefit more from a certain kind of systemic therapy. Dermatologists should take the extent and kind of childhood trauma into consideration when they decide about the appropriate dermatological treatment option in these patients. Above, childhood trauma and acute stress experiences should be carefully assessed, although psoriasis severity decreased and skin-related quality of life increased significantly under dermatological treatment. Patients with high levels of childhood/perceived acute stress should be offered an adjunct psychological support to prevent the evolvement of clinically relevant anxiety/depression.

## Data Availability Statement

The datasets generated for this study are available on request to the corresponding author.

## Ethics Statement

Ethical approval was received by the local Ethic Committee of the Technische Universität Dresden. The study was realized in accordance with the Declaration of Helsinki. Enrollment and examinations took place after the participants had given written informed consent for study participation.

## Author Contributions

G-BW, KW, and SB planned and conceptualized the study. G-BW collected the data and supervised data collection in collaboration with SA and SB. G-BW and AB analyzed the data. G-BW, EP, and KW wrote the manuscript. SA, SB, and EP supervised the study with their dermatological expertise. All authors reviewed the manuscript critically.

## Funding

We thank the foundation Robert-Pfleger-Stiftung for supporting the present study with a research fund (Fonds Number: 060_4967).

## Conflict of Interest

The authors declare that the research was conducted in the absence of any commercial or financial relationships that could be construed as a potential conflict of interest.

## Publisher's Note

All claims expressed in this article are solely those of the authors and do not necessarily represent those of their affiliated organizations, or those of the publisher, the editors and the reviewers. Any product that may be evaluated in this article, or claim that may be made by its manufacturer, is not guaranteed or endorsed by the publisher.

## Supplementary Material

Supplementary Figure S1 Kind of dermatological treatment applied in the present study after starting a new treatment episode.

Supplementary Figure S2 Frequency of a current or life-time SCID-I diagnosis in the present sample of PSO (*n* = 83).

Supplementary Figure S3a Course of (self-rated) psoriasis severity (PASI, SAPASI, BSA) in PSO (*n* = 83) from T1 to T2. All changes were highly significant at *p* ≤ .001. BSA = Body Surface Area; PASI = Psoriasis Area and Severity Index; SAPASI = Self-administered Psoriasis Area and Severity Index.

Supplementary Figure S3b Course of dermatology life quality index (DLQI), perceived stress (PSS) and anxiety/depression (HADS) in PSO (*n* = 83) from T1 to T2. DLQI changed at *p* ≤ .001, PSS at *p* ≤ .05. DLQI = Dermatology Life Quality Index; HADS = Hospital Anxiety and Depression Scale; PSS = Perceived Stress Scale.

Supplementary Figure S4 Displayed are the data for visualizing the conditional effect of the predictor Delta SAPASI on Delta DLQI, depending on the perceived stress (PSS) at T1. Z-standardized values were used. PSS = Perceived Stress Scale; SAPASI Self-administered Psoriasis Area and Severity Index.

Supplementary Table S1 Drop-Out analysis. Comparison between patients with psoriasis (PSO) (included in the final analyses) and drop outs (not followed-up at T2). For continuous variables medians and interquartile ranges (IQR) are presented. Bold values indicate significance at *p* ≤ 0.05.

Supplementary Table S2 Correlation analyses between CTQ subscales and psoriasis severity (PASI, SAPASI, BSA). Displayed are Spearman's rho correlation coefficients and *p*-values (in brackets).

Supplementary Table S3a Results of the hierarchical regression analysis to assess the effect of the variables age, gender, Delta SAPASI (step 1), PSS ‘perceived stress' at T1 (step 2) and the interaction Delta SAPASI (T1) × PSS ‘perceived stress' (step 3) on the treatment outcome (Delta DLQI). Sample size: *n* = 52 patients with complete data. Bold values indicate significance at *p* ≤ 0.05.

Supplementary Table S3b Results of the hierarchical regression analysis to assess the effect of the variables age, gender, Delta SAPASI (step 1), CTQ total at T1 (step 2) and the interaction Delta SAPASI (T1) × CTQ total (step 3) on the treatment outcome (Delta DLQI). Sample size: *n* = 58 patients with complete data. Bold values indicate significance at *p* ≤ 0.05.

Supplementary Table S4a Results of the hierarchical regression analysis to assess the effect of the variables age, gender, SAPASI (T1) (step 1), PSS ‘perceived stress' at T1 (step 2) and the interaction SAPASI (T1) × PSS ‘perceived stress' (step 3) on the treatment outcome (Delta SAPASI). Sample size: *n* = 52 patients with complete data. Bold values indicate significance at *p* ≤ 0.05.

Supplementary Table S4b Results of the hierarchical regression analysis to assess the effect of the variables age, gender, SAPASI (T1) (step 1), CTQ total at T1 (step 2) and the interaction SAPASI (T1) × CTQ total (step 3) on the treatment outcome (Delta SAPASI). Sample size: *n* = 58 patients with complete data. Bold values indicate significance at *p* ≤ 0.05.

Supplementary Table S5 Results of the moderator analysis according to the Macro Modell Process by Hayes, using *multiple imputation for missing data*. Delta SAPASI was included as independent variable. Treatment outcome (Delta DLQI) as dependent variable. Age and gender, were included as covariates, PSS ‘perceived stress' at T1 and CTQ total as moderators. Sample size: *n* = 83 patients. Bold values indicate significance at *p* ≤ 0.05.

Supplementary Table S6 Results of the moderator analysis according to the Macro Modell Process by Hayes, using *multiple imputation for missing data*. SAPASI at T1 was included as independent variable. Treatment outcome (Delta SAPASI) as dependent variable. Age and gender were included as covariates, PSS ‘perceived stress' at T1 and CTQ total as moderators. Sample size: *n* = 83 patients. Bold values indicate significance at *p* ≤ 0.05.
