# Supplementary figures and images for: Childhood Trauma and Psychosocial Stress Affect Treatment Outcome in Patients With Psoriasis Starting a New Treatment Episode
Source: Front Psychiatry. 2022 Apr 25;13:848708. doi: 10.3389/fpsyt.2022.848708 (PMC9083906; doi:10.3389/fpsyt.2022.848708)

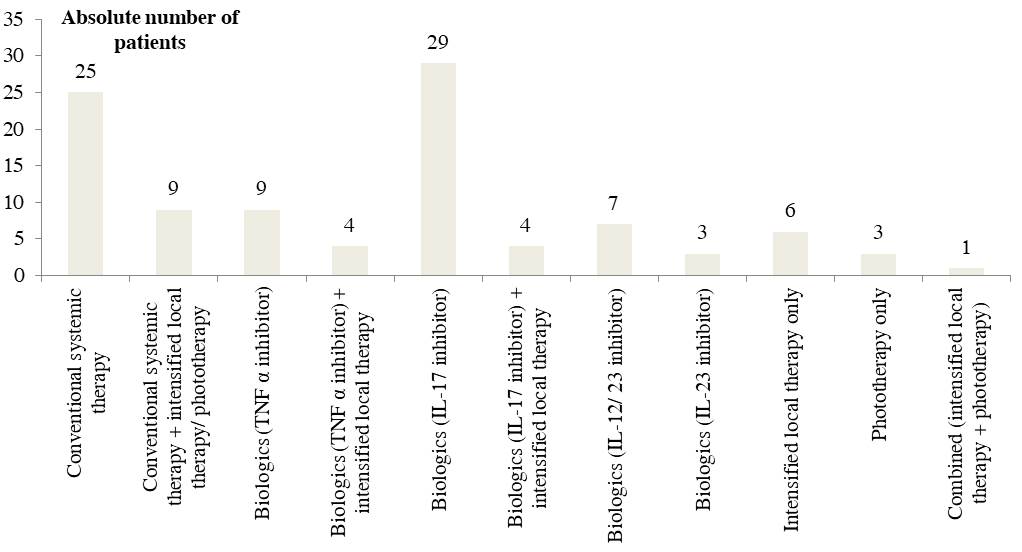

Supplement: Supplementary Figure S1 — Kind of dermatological treatment applied in the present study after starting a new treatment episode. [file Image_1.JPEG]

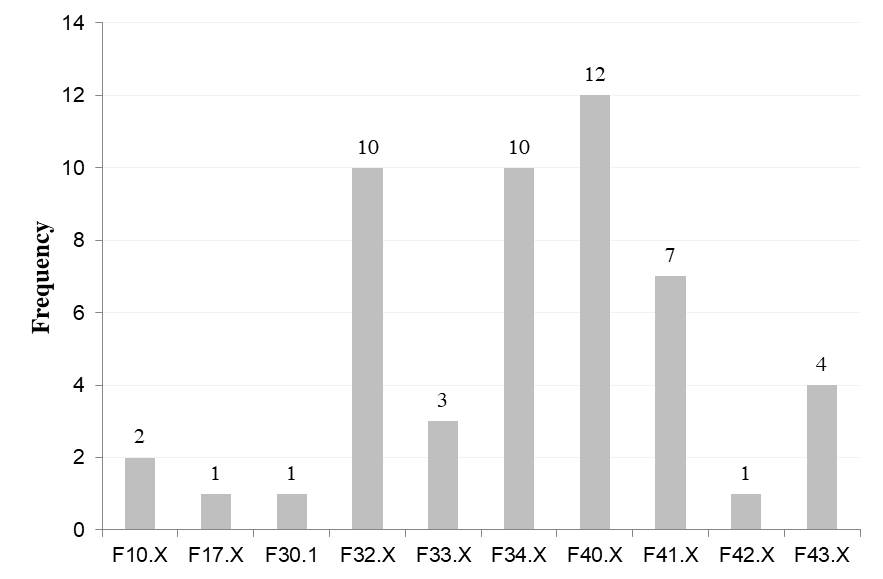

Supplement: Supplementary Figure S2 — Frequency of a current or life-time SCID-I diagnosis in the present sample of PSO (n = 83). [file Image_2.JPEG]

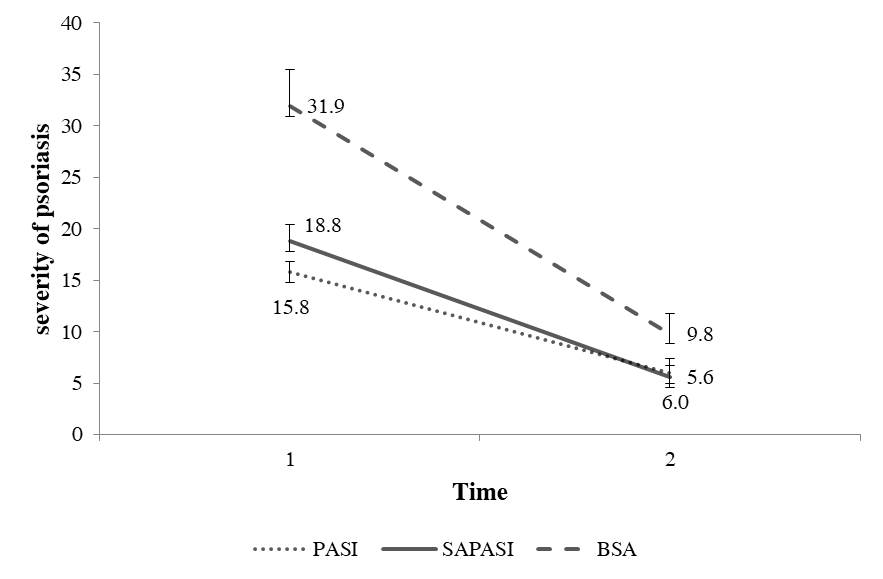

Supplement: Supplementary Figure S3a — Course of (self-rated) psoriasis severity (PASI, SAPASI, BSA) in PSO (n = 83) from T1 to T2. All changes were highly significant at p ≤ .001. BSA = Body Surface Area; PASI = Psoriasis Area and Severity Index; SAPASI = Self-administered Psoriasis Area and Severity Index. [file Image_3.JPEG]

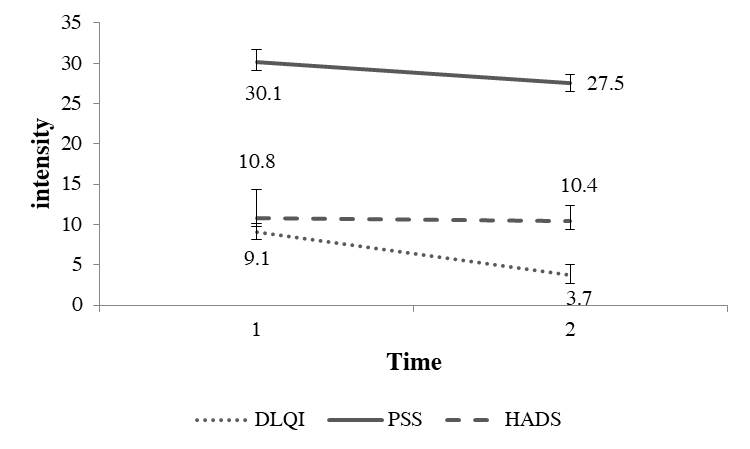

Supplement: Supplementary Figure S3b — Course of dermatology life quality index (DLQI), perceived stress (PSS) and anxiety/depression (HADS) in PSO (n = 83) from T1 to T2. DLQI changed at p ≤ .001, PSS at p ≤ .05. DLQI = Dermatology Life Quality Index; HADS = Hospital Anxiety and Depression Scale; PSS = Perceived Stress Scale. [file Image_4.JPEG]

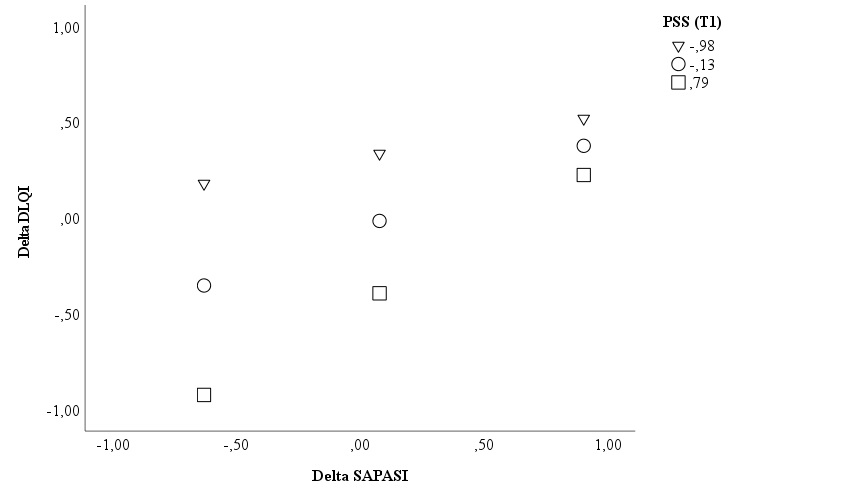

Supplement: Supplementary Figure S4 — Displayed are the data for visualizing the conditional effect of the predictor Delta SAPASI on Delta DLQI, depending on the perceived stress (PSS) at T1. Z-standardized values were used. PSS = Perceived Stress Scale; SAPASI Self-administered Psoriasis Area and Severity Index. [file Image_5.JPEG]
