# Supplementary material for: Childhood Trauma and Psychosocial Stress Affect Treatment Outcome in Patients With Psoriasis Starting a New Treatment Episode
Source: Front Psychiatry. 2022 Apr 25;13:848708. doi: 10.3389/fpsyt.2022.848708 (PMC9083906; doi:10.3389/fpsyt.2022.848708)
Supplement: Supplementary Table S1 — Drop-Out analysis. Comparison between patients with psoriasis (PSO) (included in the final analyses) and drop outs (not followed-up at T2). For continuous variables medians and interquartile ranges (IQR) are presented. Bold values indicate significance at p ≤ 0.05. [file Table_1.docx]

**Supplementary Material**

**Table S1:** Drop-Out analysis. Comparison between patients with psoriasis (PSO) (included in the final analyses) and drop outs (not followed-up at T2). For continuous variables medians and interquartile ranges (IQR) are presented.

|  | **PSO (included)**  **n = 83** | **Drop Outs**  **n = 53** | **t/ H/ U/ χ² (p)** |
| --- | --- | --- | --- |
| **Sociodemographic variables** | | | |
| men, n (%)  women, n (%) | 52 (62.7)  31 (37.3) | 31 (58.5)  22 (41.5) | χ² = .235 (.628) |
| age, years,  median (IQR) | 53.7 (37.8-62.5) | 51.4 (35.3-61.2) | U = 2073.000 (.572) |
| Family status, n (%)**^a^**  single  married  cohabited | 15 (18.1)  36 (43,4)  32 (38.6) | 13 (24.5)  23 (43.4)  14 (26.4) | χ² = 2.912  (.233) |
| Education, n (%)**^b^**  < 10 yrs  = 10 yrs  > 10 yrs | 13 (15.7)  52 (62.7)  18 (21.7) | 7 (13.2)  21 (39.6)  15 (28.3) | χ² = 2.823 (.244) |
| **Psychopathological variables** | | | |
| CTQ total score**^c^** | 15.5 (12.0-23.8) | 18.0 (13.0, 26.0) | U = 1445.000 (.179) |
| CTQ emotional abuse**^d^** | .0 (.0-3.0) | 1.0 (.0-4.0) | U = 1433.500 (.234) |
| CTQ physical abuse**^e^** | .0 (.0-1.0) | .0 (.0-2.0) | U = 1479.000 (.197) |
| CTQ sexual abuse**^f^** | .0 (.0-.0) | .0 (.0-.0) | U = 1582.000 (.302) |
| CTQ emotional neglect**^g^** | 4.0 (1.0-7.5) | 5.0 (2.0-9.0) | U = 1528.000 (.311) |
| CTQ physical neglect**^h^** | 1.0 (.0-3.5) | 2.0 (.0-4.0) | U = 1468.500 (.321) |
| MHF itching/ scratching (T1)**^i^** | 20.0 (15.0-26.0) | 22.0 (18.0-26.3) | U = 1745.500 (.125) |
| HADS anxiety (T1)**^i^** | 5.0 (2.0-8.0) | 6.0 (4.8-9.0) | U = 1514.000 (**.009**)~ |
| HADS depression (T1)**^i^** | 5.0 (2.0-8.0) | 6.5 (4.0-9.0) | U = 1661.000 (.054) |
| PSS (T1)**^j^** | 30.0 (25.0-34.0) | 33.0 (26.5-38.0) | U = 1169.500 (.075) |
| DLQI (T1)**^k^** | 7.0 (3.0-13.0) | 13.0 (7.0-16.0) | U = 1511.500 (**.005**)° |
| **Medical variables** | | | |
| SAPASI**^l^** | 15.6 (7.4-24.2) | 15.2 (10.2-30.7) | U = 1537.000 (.573) |
| BSA**^m^** | 18.0 (9.0-40.0) | 21.0 (11.0-47.5) | U = 1798.000 (.431) |
| PASI**^n^** | 13.8 (10.4-20.1) | 13.9 (8.4-21.1) | U = 1759.500 (.797) |
| number of body areas affected**^o^** | 8.0 (5.5 -10.0) | 9.0 (7.0-11.5) | U = 1601.000 (.187) |
| Duration of psoriasis**^p^** | 18.0 (6.7-27.2) | 16.1 (3.6-25.2) | U = 1962.000 (.376) |
| Onset of psoriasis (age)**^p^** | 30.1 (17.4-41.4) | 30.7 (17.6-42.8) | U = 2041.500 (.598) |

**^a^**Drop Outs: 3 missing values; **^b^**Drop Outs: 10 missing values; **^c^**Drop Outs: 6 missing values, Included: 11 missing values; **^d^**Drop Outs: 7 missing values, Included: 12 missing values; **^e^**Drop Outs: 7 missing values, Included: 10 missing values; **^f^**Drop Outs: 6 missing values, Included: 12 missing values; **^g^**Drop Outs: 6 missing values, Included: 10 missing values; **^h^**Drop Outs: 8 missing values, Included: 10 missing values; **^i^**Drop Outs: 3 missing values; **^j^**Drop Outs: 8 missing values, Included: 18 missing values; **^k^**Drop Outs: 2 missing values; **^l^**Drop Outs: 12 missing values, Included: 3 missing values; **^m^**Drop Outs: 4 missing values, Included: 3 missing values; **^n^**Included/ Drop Outs: 6 missing values; **^o^**Drop Outs: 7 missing values, Included: 2 missing values; **^p^**Drop Outs: 1 missing value; BSA = Body Surface Area; DLQI = Dermatology Life Quality Index; HADS = Hospital Anxiety and Depression Scale; IQR = Interquartile Range; MHF = Marburg Skin Questionnaire/ Marburger Haut-Fragebogen; PASI = Psoriasis Area and Severity Index; PSS = Perceived Stress Scale; SAPASI = Self-Administered Psoriasis Area and Severity Index; Bold values: ~significance at Bonferroni-corrected p-value ≤ .03; °significance at p-value ≤ .05
