# Supplementary material for: Childhood Trauma and Psychosocial Stress Affect Treatment Outcome in Patients With Psoriasis Starting a New Treatment Episode
Source: Front Psychiatry. 2022 Apr 25;13:848708. doi: 10.3389/fpsyt.2022.848708 (PMC9083906; doi:10.3389/fpsyt.2022.848708)
Supplement: Supplementary Table S2 — Correlation analyses between CTQ subscales and psoriasis severity (PASI, SAPASI, BSA). Displayed are Spearman's rho correlation coefficients and p-values (in brackets). [file Table_2.docx]

**Supplementary Material**

**Table S2:** Correlation analyses between CTQ subscales and psoriasis severity (PASI, SAPASI, BSA). Displayed are Spearman´s rho correlation coefficients and p-values (in brackets).

|  | ***T1*** | | | ***T2*** | | |
| --- | --- | --- | --- | --- | --- | --- |
| *Spearman´s rho (p values)* | ***PASI*** | ***SAPASI*** | ***BSA*** | ***PASI*** | ***SAPASI*** | ***BSA*** |
| ***CTQ total*** | *-.041 (.744)*^a^ | *.083*  *(.495)*^d^ | *-.068*  *(.576)*^f^ | *-.155 (.492)*^h^ | *-.098*  *(.454)*^j^ | *-.109*  *(.362)*^m^ |
| ***CTQ emotional abuse*** | *-.063 (.619)*^b^ | *.111*  *(.368)*^e^ | *-.122*  *(.318)*^d^ | *-.146 (.529)*^i^ | *-.161*  *(.218)*^k^ | *-.121*  *(.315)*^g^ |
| ***CTQ physical abuse*** | *-.015 (.903)*^c^ | *.148 (.222)*^f^ | *-.026*  *(.828)*^g^ | *-.152 (.500)*^h^ | *-.105 (.416)*^l^ | *-.119*  *(.318)*^n^ |
| ***CTQ sexual abuse*** | *.047 (.709)*^b^ | *.112*  *(.362)*^e^ | *-.052*  *(.671)*^d^ | *-.025 (.910)*^h^ | *.124*  *(.345)*^k^ | *.121*  *(.314)*^g^ |
| ***CTQ emotional neglect*** | *.013 (.918)*^c^ | *.149*  *(.217)*^f^ | *.028*  *(.817)*^g^ | *-.032 (.889)*^h^ | *-.005*  *(.966)*^l^ | *-.015*  *(.897)*^n^ |
| ***CTQ physical neglect*** | *.114 (.357)*^c^ | *.149*  *(.217)*^f^ | *.083*  *(.491)*^g^ | *-.073 (.746)*^h^ | *.052*  *(.687)*^l^ | *.051*  *(.669)*^n^ |

^a^ n = 66, ^b^ n = 65, ^c^ n = 67, ^d^ n = 69, ^e^ n = 68, ^f^ n = 70, ^g^ n = 71, ^h^ n = 22, ^i^ n = 21, ^j^n = 61, ^k^n = 60, ^l^n = 62, ^m^n = 72, ^n^n = 73; BSA = Body Surface Area; CTQ = Childhood Trauma Questionnaire; IQR = Interquartile Range; PASI = Psoriasis Area and Severity Index; SAPASI = Self-administered Psoriasis Area and Severity Index
