# Supplementary material for: Childhood Trauma and Psychosocial Stress Affect Treatment Outcome in Patients With Psoriasis Starting a New Treatment Episode
Source: Front Psychiatry. 2022 Apr 25;13:848708. doi: 10.3389/fpsyt.2022.848708 (PMC9083906; doi:10.3389/fpsyt.2022.848708)
Supplement: Supplementary Table S3a — Results of the hierarchical regression analysis to assess the effect of the variables age, gender, Delta SAPASI (step 1), PSS ‘perceived stress' at T1 (step 2) and the interaction Delta SAPASI (T1) × PSS ‘perceived stress' (step 3) on the treatment outcome (Delta DLQI). Sample size: n = 52 patients with complete data. Bold values indicate significance at p ≤ 0.05. [file Table_3.docx]

**Supplementary Material**

**Table S3a:** Results of the hierarchical regression analysis to assess the effect of the variables age, gender, Delta SAPASI (step 1), PSS ‘perceived stress’ at T1 (step 2) and the interaction Delta SAPASI (T1) x PSS ‘perceived stress’ (step 3) on the treatment outcome (Delta DLQI). Sample size: n = 52 patients with complete data

|  | **Beta** | **T** | **F** | **R²** | **Adj. R²** | **Delta R²** | **Delta F** | **p** | **Confidence Interval** |
| --- | --- | --- | --- | --- | --- | --- | --- | --- | --- |
| *Step 1* |  |  | 5.272 | .248 | .201 | .248 | 5.272 | **.003** |  |
| Age | -.085 | -.636 |  |  |  |  |  | .527 | -.355, .184 |
| Gender | .158 | 1.259 |  |  |  |  |  | .214 | -.202, .878 |
| Delta SAPASI | .498 | 3.739 |  |  |  |  |  | **<.001** | .246, .817 |
| *Step 2* |  |  | 8.452 | .418 | .369 | .171 | 13.781 | **<.001** |  |
| Age | -.054 | -.459 |  |  |  |  |  | .648 | -.295, .186 |
| Gender | .091 | .803 |  |  |  |  |  | .426 | -.292 .681 |
| Delta SAPASI | .459 | 3.866 |  |  |  |  |  | **<.001** | .235, .745 |
| PSS ‚perceived stress‘ (T1) | -.420 | -3.712 |  |  |  |  |  | **<.001** | -.668, -.199 |
| *Step 3* |  |  | 8.466 | .479 | .423 | .061 | 5.374 | **<.001** |  |
| Age | -.022 | -.196 |  |  |  |  |  | .845 | -.254, .209 |
| Gender | .047 | .430 |  |  |  |  |  | .669 | -.372, .573 |
| Delta SAPASI | -.540 | -1.212 |  |  |  |  |  | .232 | -1.536, .381 |
| PSS ‘perceived stress’ (T1) | -.171 | -1.121 |  |  |  |  |  | .268 | -.493, .140 |
| SAPASI T1 x PSS ‚perceived stress‘ (T1) | 1.066 | 2.318 |  |  |  |  |  | **.025** | .144, 2.045 |

DLQI = Dermatology Life Quality Index; PSS = Perceived Stress Scale

**Table S3b:** Results of the hierarchical regression analysis to assess the effect of the variables age, gender, Delta SAPASI (step 1), CTQ total at T1 (step 2) and the interaction Delta SAPASI (T1) x CTQ total (step 3) on the treatment outcome (Delta DLQI). Sample size: n = 58 patients with complete data

|  | **Beta** | **T** | **F** | **R²** | **Adj. R²** | **Delta R²** | **Delta F** | **p** | **Confidence Interval** |
| --- | --- | --- | --- | --- | --- | --- | --- | --- | --- |
| *Step 1* |  |  | 4.043 | .183 | .138 | .183 | 4.043 | **.012** |  |
| Age | -.022 | -.172 |  |  |  |  |  | .864 | -.276, .232 |
| Gender | .100 | .811 |  |  |  |  |  | .421 | -.314, .741 |
| Delta SAPASI | .422 | 3.284 |  |  |  |  |  | **.002** | .168, .694 |
| *Step 2* |  |  | 4.074 | .235 | .177 | .052 | 3.585 | **.006** |  |
| Age | -.031 | -.247 |  |  |  |  |  | .806 | -.279, .218 |
| Gender | .069 | .567 |  |  |  |  |  | .573 | -.373 .668 |
| Delta SAPASI | .352 | 2.692 |  |  |  |  |  | **.009** | .092, .628 |
| CTQ total (T1) | -.241 | -1.893 |  |  |  |  |  | .064 | -.510, .015 |
| *Step 3* |  |  | 3.233 | .237 | .164 | .002 | .136 | **.013** |  |
| Age | -.037 | -.290 |  |  |  |  |  | .773 | -.289, .216 |
| Gender | .060 | .478 |  |  |  |  |  | .635 | -.408, .663 |
| Delta SAPASI | .440 | 1.615 |  |  |  |  |  | .112 | -.109, 1.008 |
| CTQ total (T1) | -.215 | -1.473 |  |  |  |  |  | .147 | -.522, .080 |
| Delta SAPASI x CTQ total (T1) | .094 | .368 |  |  |  |  |  | .714 | -.417, .604 |

CTQ = Childhood Trauma Questionnaire; DLQI = Dermatology Life Quality Index
