# Supplementary material for: Childhood Trauma and Psychosocial Stress Affect Treatment Outcome in Patients With Psoriasis Starting a New Treatment Episode
Source: Front Psychiatry. 2022 Apr 25;13:848708. doi: 10.3389/fpsyt.2022.848708 (PMC9083906; doi:10.3389/fpsyt.2022.848708)
Supplement: Supplementary Table S4a — Results of the hierarchical regression analysis to assess the effect of the variables age, gender, SAPASI (T1) (step 1), PSS ‘perceived stress' at T1 (step 2) and the interaction SAPASI (T1) × PSS ‘perceived stress' (step 3) on the treatment outcome (Delta SAPASI). Sample size: n = 52 patients with complete data. Bold values indicate significance at p ≤ 0.05. [file Table_4.docx]

**Supplementary Material**

**Table S4a:** Results of the hierarchical regression analysis to assess the effect of the variables age, gender, SAPASI (T1) (step 1), PSS ‘perceived stress’ at T1 (step 2) and the interaction SAPASI (T1) x PSS ‘perceived stress’ (step 3) on the treatment outcome (Delta SAPASI). Sample size: n = 52 patients with complete data

|  | **Beta** | **T** | **F** | **R²** | **Adj. R²** | **Delta R²** | **Delta F** | **p** | **Confidence Interval** |
| --- | --- | --- | --- | --- | --- | --- | --- | --- | --- |
| *Step 1* |  |  | 15.373 | .490 | .458 | .490 | 15.373 | **<.001** |  |
| Age | .172 | 1.608 |  |  |  |  |  | .114 | -.041, .365 |
| Gender | -.047 | -.456 |  |  |  |  |  | .650 | -.511, .322 |
| SAPASI (T1) | -.635 | -5.937 |  |  |  |  |  | **<.001** | -.949, -.469 |
| *Step 2* |  |  | 11.583 | .496 | .454 | .006 | .599 | **<.001** |  |
| Age | .175 | 1.632 |  |  |  |  |  | .109 | -.038, .369 |
| Gender | -.060 | -.571 |  |  |  |  |  | .571 | -.544, .304 |
| SAPASI (T1) | -.635 | -5.909 |  |  |  |  |  | **<.001** | -.950, -.467 |
| PSS ‚perceived stress‘ (T1) | -.081 | -.774 |  |  |  |  |  | .443 | -.282, .125 |
| *Step 3* |  |  | 11.176 | .548 | .499 | .052 | 5.303 | **<.001** |  |
| Age | .208 | 2.002 |  |  |  |  |  | .051 | -.001, .393 |
| Gender | -.085 | -.836 |  |  |  |  |  | .407 | -.578, .239 |
| SAPASI (T1) | .452 | .936 |  |  |  |  |  | .354 | -.581, 1.590 |
| PSS ‚perceived stress‘ (T1) | .409 | 1.738 |  |  |  |  |  | .089 | -.063, .853 |
| SAPASI T1 x PSS ‚perceived stress‘ (T1) | -1.210 | -2.303 |  |  |  |  |  | **.026** | -2.514, -.169 |

CTQ = Childhood Trauma Questionnaire; PSS = Perceived Stress Scale; SAPASI = Self-administered Psoriasis Area and Severity Index

**Table S4b:** Results of the hierarchical regression analysis to assess the effect of the variables age, gender, SAPASI (T1) (step 1), CTQ total at T1 (step 2) and the interaction SAPASI (T1) x CTQ total (step 3) on the treatment outcome (Delta SAPASI). Sample size: n = 58 patients with complete data

|  | **Beta** | **T** | **F** | **R²** | **Adj. R²** | **Delta R²** | **Delta F** | **p** | **Confidence Interval** |
| --- | --- | --- | --- | --- | --- | --- | --- | --- | --- |
| *Step 1* |  |  | 20.983 | .538 | .513 | .538 | 20.983 | **<.001** |  |
| Age | .146 | 1.542 |  |  |  |  |  | .129 | -.042, .324 |
| Gender | -.029 | -.312 |  |  |  |  |  | .757 | -.449, .329 |
| SAPASI (T1) | -.691 | -7.301 |  |  |  |  |  | **<.001** | -.963, -.548 |
| *Step 2* |  |  | 18.972 | .589 | .558 | .051 | 6.512 | **<.001** |  |
| Age | .122 | 1.342 |  |  |  |  |  | .185 | -.058, .292 |
| Gender | -.058 | -.656 |  |  |  |  |  | .515 | -.496, .252 |
| SAPASI (T1) | -.675 | -7.474 |  |  |  |  |  | **<.001** | -.937, -.541 |
| CTQ total (T1) | -.229 | -2.552 |  |  |  |  |  | **.014** | -.049, -.411 |
| *Step 3* |  |  | 18.635 | .642 | .607 | .053 | 7.699 | **<.001** |  |
| Age | .093 | 1.087 |  |  |  |  |  | .282 | -.076, .256 |
| Gender | -.066 | -.783 |  |  |  |  |  | .437 | -.490, .215 |
| SAPASI (T1) | -1.229 | -5.664 |  |  |  |  |  | **<.001** | -1.821, -.868 |
| CTQ total (T1) | .156 | .961 |  |  |  |  |  | .341 | -.171, .485 |
| SAPASI T1 x CTQ total (T1) | -.681 | -2.775 |  |  |  |  |  | **.008** | -1.219, -.196 |

CTQ = Childhood Trauma Questionnaire; PSS = Perceived Stress Scale; SAPASI = Self-administered Psoriasis Area and Severity Index
