# Supplementary material for: Childhood Trauma and Psychosocial Stress Affect Treatment Outcome in Patients With Psoriasis Starting a New Treatment Episode
Source: Front Psychiatry. 2022 Apr 25;13:848708. doi: 10.3389/fpsyt.2022.848708 (PMC9083906; doi:10.3389/fpsyt.2022.848708)
Supplement: Supplementary Table S5 — Results of the moderator analysis according to the Macro Modell Process by Hayes, using multiple imputation for missing data. Delta SAPASI was included as independent variable. Treatment outcome (Delta DLQI) as dependent variable. Age and gender, were included as covariates, PSS ‘perceived stress' at T1 and CTQ total as moderators. Sample size: n = 83 patients. Bold values indicate significance at p ≤ 0.05. [file Table_5.docx]

**Supplementary Material**

**Table S5:** Results of the moderator analysis according to the Macro Modell Process by Hayes, using *multiple imputation for missing data*. Delta SAPASI was included as independent variable. Treatment outcome (Delta DLQI) as dependent variable. Age and gender, were included as covariates, PSS ‘perceived stress’ at T1 and CTQ total as moderators. Sample size: n = 83 patients

|  | **Beta** | **t** | **Lower 95% CI** | **Upper 95% CI** | **p** |  | **Beta** | **t** | **Lower 95% CI** | **Upper 95% CI** | **p** |
| --- | --- | --- | --- | --- | --- | --- | --- | --- | --- | --- | --- |
| **Delta DLQI** | | | | | | | | | | | |
| Age | .054 | .594 | .554 | -.126 | .233 | Age | .028 | .283 | -.171 | .228 | .778 |
| Gender | .344 | 1.874 | -.022 | .709 | .065 | Gender | .380 | 1.836 | -.032 | .792 | .070 |
| Delta SAPASI | .443 | 4.926 | .264 | .621 | **<.001** | Delta SAPASI | .415 | 4.095 | .213 | .617 | **< .001** |
| PSS ‚perceived stress‘ (T1) | -.274 | -3.057 | -.453 | -.096 | **.003** | CTQ total (T1) | -.163 | -1.583 | -.368 | .042 | .117 |
| Delta SAPASI x PSS ‚perceived stress‘ (T1) | .420 | 3.725 | .196 | .645 | **<.001** | Delta SAPASI x CTQ total (T1) | .031 | .314 | -.163 | .224 | .754 |
| n = 83, F(5,77) = 10.008, p < .001, R² = 39.4 | | | | | | n = 83, F(5,77) = 5.334, p < .001, R² = 25.7 | | | | | |

CTQ = Childhood Trauma Questionnaire; DLQI = Dermatology Life Quality Index; SAPASI = Self-administered Psoriasis Area and Severity Index
