# Supplementary material for: Childhood Trauma and Psychosocial Stress Affect Treatment Outcome in Patients With Psoriasis Starting a New Treatment Episode
Source: Front Psychiatry. 2022 Apr 25;13:848708. doi: 10.3389/fpsyt.2022.848708 (PMC9083906; doi:10.3389/fpsyt.2022.848708)
Supplement: Supplementary Table S6 — Results of the moderator analysis according to the Macro Modell Process by Hayes, using multiple imputation for missing data. SAPASI at T1 was included as independent variable. Treatment outcome (Delta SAPASI) as dependent variable. Age and gender were included as covariates, PSS ‘perceived stress' at T1 and CTQ total as moderators. Sample size: n = 83 patients. Bold values indicate significance at p ≤ 0.05. [file Table_6.docx]

**Supplementary Material**

**Table S6:** Results of the moderator analysis according to the Macro Modell Process by Hayes, using *multiple imputation for missing data*. SAPASI at T1 was included as independent variable. Treatment outcome (Delta SAPASI) as dependent variable. Age and gender were included as covariates, PSS ‘perceived stress’ at T1 and CTQ total as moderators. Sample size: n = 83 patients

|  | **Beta** | **t** | **Lower 95% CI** | **Upper 95% CI** | **p** |  | **Beta** | **t** | **Lower 95% CI** | **Upper 95% CI** | **p** |
| --- | --- | --- | --- | --- | --- | --- | --- | --- | --- | --- | --- |
| **Delta SAPASI** | | | | | | | | | | | |
| Age | .069 | 1.021 | -.065 | .202 | .311 | Age | .030 | .479 | -.094 | .153 | .633 |
| Gender | -.169 | -1.231 | -.442 | .104 | .222 | Gender | -.192 | -1.510 | -.445 | .061 | .135 |
| SAPASI (T1) | -.804 | -12.003 | -.938 | -.671 | **<.001** | SAPASI (T1) | -.750 | -12.029 | -.874 | -.625 | **<.001** |
| PSS ‚perceived stress‘ (T1) | -.020 | -.300 | -.156 | .115 | .765 | CTQ total (T1) | -.143 | -2.307 | -.266 | -.020 | **.024** |
| SAPASI T1 x PSS ‚perceived stress‘ (T1) | -.128 | -1.524 | -.296 | .039 | .132 | SAPASI T1 x CTQ total (T1) | -.258 | -3.621 | -.400 | -.116 | **<.001** |
| n = 83, F(5,77) = 29.965, p < .001, R² = 66.1 | | | | | | n = 83, F(5,77) = 38.627, p < .001, R² = 71.5 | | | | | |

CTQ = Childhood Trauma Questionnaire; SAPASI = Self-administered Psoriasis Area and Severity Index
